# EPR spectroscopy reveals antioxidant manganese defenses in the Lyme disease pathogen *Borrelia burgdorferi*

**DOI:** 10.1128/mbio.02824-25

**Published:** 2025-11-13

**Authors:** Andrés F. Londoño, Ajay Sharma, Venkatesan Kathiresan, Jared Sealy, Robert P. Volpe, Cene Gostinčar, Utpal Pal, J. Stephen Dumler, Brian M. Hoffman, Michael J. Daly

**Affiliations:** 1 School of Medicine, Uniformed Services University of the Health Sciences (USUHS) 1685, Bethesda, Maryland, USA; 2 Henry M. Jackson Foundation for the Advancement of Military Medicine 44069 , Bethesda, Maryland, USA; 3 Department of Chemistry, Northwestern University 309924, Evanston, Illinois, USA; 4 The Cooper Union for the Advancement of Science 5937, New York, New York, USA; 5 Department of Biology, Biotechnical Faculty, University of Ljubljana 196560, Ljubljana, Slovenia; 6 Department of Veterinary Medicine, University of Maryland 212460 , College Park, Maryland, USA; NYU Langone Health, New York, New York, USA

**Keywords:** *Borrelia*, *Deinococcus*, *Lactobacillus*, *Bacillus*, ionizing radiation, ROS, MnSOD, Mn-antioxidant, Mn speciation, Mn toxicity, metabolite transport, EPR, ENDOR

## Abstract

Oxidative stress defense in aerobic bacteria relies on Mn-superoxide dismutase (MnSOD) and antioxidant Mn-metabolite complexes (H-Mn) to quench superoxide radicals (O_2_^•−^). We investigated these antioxidant systems in *Borrelia burgdorferi*, the Mn-accumulating, Fe-independent Lyme disease pathogen. Using electron paramagnetic resonance (EPR) and electron-nuclear double resonance (ENDOR) spectroscopies, we tracked Mn²^+^ partitioning between enzyme-bound (L-Mn) and metabolite-bound (H-Mn) pools in spirochetes at exponential and stationary phases. Results show that MnSOD neutralizes extracellular O_2_^•−^ generated by γ-irradiation (a model for host immune attack); H-Mn neutralizes cytoplasmic O_2_^•−^ and is a reservoir of labile Mn²^+^ for metalating Mn-dependent enzymes. MnCl_2_ supplementation in log phase *B. burgdorferi* restored radioresistance in ΔMnSOD mutants via H-Mn hyperaccumulation but induced toxicity in older, stationary phase cells as metabolites became depleted. These findings support an expanded oxidative-stress model in which H-Mn complements MnSOD and positions Mn homeostasis as a therapeutic target. Our approach highlights the utility of EPR and ENDOR in studying Mn-dependent pathogens.

**IMPORTANCE:**

We employed electron paramagnetic resonance and electron-nuclear double resonance spectroscopies of Mn²^+^ in intact *Borrelia burgdorferi* supplemented with MnCl_2_ to track changes in the amounts of enzyme-bound Mn and substitutionally labile, antioxidant Mn-metabolite complexes. We measured the spirochete’s survivability to acute γ-irradiation, which simulates the respiratory burst of O_2_^•−^ deployed as a critical weapon in the host’s innate immune response. While Mn-superoxide dismutase (MnSOD) has classically been viewed as the main defense against oxidative damage in *B. burgdorferi*, our study demonstrates that antioxidant Mn^2+^ complexes with the metabolite components of H-Mn play a crucial antioxidant role, particularly when MnSOD is deficient. However, *B. burgdorferi’s* inability to safely store excess Mn in metabolite-depleted cells highlights novel metabolic vulnerabilities that could be exploited for managing Lyme disease.

## INTRODUCTION

The essential role of manganese (Mn) in antioxidant defense involves both enzymatic superoxide dismutase (MnSOD) and nonenzymatic Mn²^+^-antioxidant metabolite complexes (H-Mn), which are characterized by electron paramagnetic resonance (EPR) spectroscopies (1, 2). These two Mn-dependent components have been the focus of research aimed at understanding their respective contributions to mitigating the cytotoxic effects of reactive oxygen species (ROS) generated by radiation, desiccation, and cellular respiration (3–5). Manganese defends against ROS by preventing damage to proteins, including those that repair DNA double-strand breaks (DSBs). First, this is achieved by replacing Fe^2+^ (and other divalent metals) with Mn^2+^ as cofactors in enzymes, particularly SOD, which protects active sites from oxidative damage (6–10). Second, and equally important, is the labile Mn^2+^ pool—the fraction not bound to proteins—which forms antioxidant Mn^2+^ complexes with metabolites (e.g., carboxylic acids including lactate, bicarbonate, orthophosphate, amino acids, and peptides). These H-Mn antioxidant complexes form in proportion to the metabolite and Mn^2+^ concentrations, i.e., the substitutionally labile Mn^2+^ ion is an indicator of the cellular metabolite population (11–17). Significantly, the H-Mn pool includes ternary complexes of Mn^2+^ coordinated with both an orthophosphate (Pi) and an additional metabolite. While Mn-Pi alone exhibits considerable catalytic ability, Mn-Pi coordinated with peptides (e.g., in *Deinococcus radiodurans*) yields H-Mn complexes with much greater superoxide (O_2_^•−^)-scavenging efficiency than Mn-Pi alone, and comparable to MnSOD (14, 18). H-Mn antioxidants protect cytoplasmic proteins from oxidative assault, in particular preserving the DNA repair and metabolic functions needed to efficiently reassemble genomes broken by radiation and during desiccation in free-living bacteria (19). As the pool of cytoplasmic metabolites needed to form H-Mn^2+^ complexes depends on the nutritional status of the cell, radioresistance is correlated with metabolism (2, 5).

Radiation- and desiccation-resistant free-living bacteria are renowned for their capacity to accumulate Mn over iron (Fe) and display disproportionately high intracellular Mn/Fe concentration ratios compared to those of radiation-sensitive bacteria (20, 21). In particular, Mn-accumulating free-living bacteria do not, as a rule, accumulate significant cytoplasmic Fe (22), which would carry out the harmful (i) Fenton reaction, which generates ultra-short-lived hydroxyl radicals (HO^•^), and (ii) Haber-Weiss reaction, which generates long-lived O_2_^•−^ radicals, through reaction with hydrogen peroxide (H_2_O_2_), the end product of reactions of MnSOD and Mn antioxidants with O_2_^•−^ (4). We here apply ionizing radiation in the form of gamma rays whose acute generation of ROS (HO^•^, O_2_^•−^, H_2_O_2_) is mechanistically understood (23). Critically, while redox cycling of Mn^2+^ efficiently catalyzes scavenging of O_2_^•−^ radicals, unlike Fe^2+^, it is not reactive with H_2_O_2_, and this helps prevent the Fe-catalyzed formation of additional O_2_^•−^ (11, 12, 14, 22).

While the Mn defenses against ROS in free-living terrestrial Mn-accumulating bacteria like *Deinococcus radiodurans*, *Lactobacillus plantarum*, and *Bacillus subtilis* have been characterized extensively (4, 5, 24, 25), their function in pathogenic bacteria remains less clear—indeed, manganese generally has been assigned little importance in pathogens other than as a cofactor for some ROS detoxifying enzymes and as a target of nutritional immunity (9, 26–29). While the habitats of free-living bacteria are prone to cycles of desiccation and rehydration, as well as solar irradiation, both of which generate bactericidal ROS (23), Mn-accumulating pathogens such as *Borrelia burgdorferi* spirochetes are shielded within their hosts from both drying and solar ultraviolet (UV) radiation. However, they face unique challenges from the hostile ROS generated by the host’s native immune response (30–33). In particular, *B. burgdorferi*, the tick-borne causative agent of Lyme disease, must navigate the hostile milieus of both tick and mammalian hosts (34). The host nonspecific innate immune system prominently employs oxidative stress by O_2_^•−^ as a defense against microbial agents (35–37), which is distinct from the environmental oxidative effects of desiccation or radiation experienced by free-living bacteria in dry-climate habitats (21).

The limited ability of O_2_^•−^ to cross lipid membranes dictates how we understand the distinction between host-derived and environmental assaults by O_2_^•−^, as well as the strategic localization and roles of MnSOD and Mn antioxidants (38). Consequently, environmental O_2_^•−^ assault at the cell surface should not create a need for cytoplasmic SOD activity in bacteria (39, 40). Instead, bacterial pathogens facing extracellular attack by nonspecific host immune responses that generate O_2_^•−^ radicals require MnSOD at the cell periphery as a defense mechanism for survival. Likewise, during acute aerobic γ-irradiation, unlimited O_2_^•−^ radicals generated outside cells attack their surfaces, and thus irradiation in part acts as a model of the host immune response. However, limited O_2_^•−^ also transiently accumulates inside the cytoplasm of irradiated cells and is further generated during recovery by damaged metabolic machinery. This cytoplasmic O_2_^•−^ is neutralized by H-Mn antioxidants (20, 22, 23, 41, 42). Notably, these two distinct sources of O_2_^•−^ explain why MnSOD is dispensable for radiation resistance in free-living microorganisms like *D. radiodurans* but indispensable for desiccation resistance and defense against severe external O_2_^•−^-generating agents like methyl viologen (paraquat) (5). This conceptual distinction supports the model that MnSOD at the cell surface provides the primary antioxidant defense in *Borrelia* pathogens within their natural host environments (43–45). We note, however, that MnSOD surface localization has not yet been experimentally established in either *B. burgdorferi* or *D. radiodurans* (Text S1).

Generally, the Mn-binding affinity of Mn enzymes (e.g., SodA) and other Mn proteins (e.g., DNA-binding protein from starved cells; DPS) is considerably higher than that of cellular metabolites, creating strategic competition for the cellular Mn^2+^, with abundant Mn-dependent holo-SodA able to outcompete the cellular metabolites (5, 18, 25, 46–51). Consequently, only after Mn enzymes are fully metalated does any remaining Mn^2+^ accumulate as a labile pool of H-Mn metabolite complexes, thereby enhancing cellular radiation resistance (2, 10, 18, 52). Conversely, under conditions of metabolite depletion—as cells transition from exponential to stationary state and starve in depleted media—the H-Mn content and corresponding radioresistance decrease (5, 53). In stationary phase deinococci, labile Mn^2+^ ions released from a dwindling pool of H-Mn antioxidants are stored in cytoplasmic granules enriched in Mn, Ca, and polyphosphate and mobilized by starvation-induced DPS proteins (19, 22, 49, 50, 54), as modulated via the canonical (p)ppGpp-driven stringent response to nutrient restriction (55–58). However, once the “Mn-buffering” capacity of proteins and the metabolite pool is exhausted in stationary phase cells, any excess Mn^2+^ becomes cytotoxic.

*Borrelia burgdorferi* distinguishes itself among pathogenic bacteria by its inability to accumulate iron (59, 60), which should be a distinct advantage under ionizing radiation because it eliminates Fe-catalyzed Haber-Weiss and Fenton reactions with H_2_O_2_ (10, 20, 23, 41). Instead, this multigenomic spirochete accumulates manganese, a trait suggesting that it should exhibit high radiation resistance, as observed in *D. radiodurans* (20, 27). However, contrary to expectations, *B. burgdorferi* grown in standard liquid Barbour-Stoenner-Kelly (BSK)-H medium exhibits significant radiosensitivity (45), with a survival limit even lower than that of the radiation-sensitive *Escherichia coli*, which can withstand only a few chromosomal DSBs when irradiated (2). Our recent EPR studies revealed that *B. burgdorferi* has a relatively small H-Mn population, which, along with its unique linear genome architecture, contributes to its radiosensitivity (45). Here, we expand on those findings using EPR and electron-nuclear double resonance (ENDOR) spectroscopies to investigate the complementary roles of the two antioxidant defenses—MnSOD and the H-Mn pool of Mn-metabolite complexes—in *B. burgdorferi* cells, while the manganese metabolic environment varies as the spirochetes transition from exponential to stationary phase.

EPR/ENDOR spectroscopy of cellular Mn provides a unique window into the *in vivo* dynamics of Mn speciation. Traditional biochemical approaches disrupt cells and fail to preserve crucial information about Mn^2+^ coordination, as this ion is substitutionally labile and rapidly equilibrates with a changing environment by exchanging bound ligands (25). As a result, analysis-by-isolation methods are unable to unambiguously characterize enzymatic and nonenzymatic modes of O_2_^•−^ scavenging. In contrast, EPR spectroscopy non-invasively characterizes Mn^2+^ speciation in living cells, distinguishing low-symmetry (L-Mn) enzyme complexes such as MnSOD from high-symmetry (H-Mn) metabolite complexes, while ENDOR spectroscopy identifies the ligand population of the H-Mn (1, 48, 61–64). These techniques have revealed that radiation-resistant microbes possess a high population of H-Mn antioxidant metabolite complexes, whereas radiation-sensitive cell types predominantly contain the L-Mn MnSOD; our previous EPR studies demonstrate that radiation-resistant archaea, bacteria, and eukaryotes consistently hyperaccumulate H-Mn antioxidants (2, 62). Moreover, among microorganisms that hyperaccumulate H-Mn antioxidants, polyploid prokaryotes (*Deinococcus*, *Chroococcidiopsis,* and *Halobacteria* spp.) exhibit the highest radiation resistance, as exemplified by bacteria of the genus *Deinococcus* (4–8*n*), which can survive frozen following γ-ray doses that exceed 70 kGy (65). Significantly, H-Mn^2+^ speciation remains stable even under supralethal doses of gamma radiation, and the activity of H-Mn antioxidants is not diminished by such extreme exposures (18, 48). EPR/ENDOR thus offers a robust method for investigating responses of Mn-dependent pathogens to the oxidative stresses imposed by radiation (45, 66).

To understand the behavior of Mn^2+^ oxidative defenses in the *B. burgdorferi* spirochete, the present study employs radiation survival measurements for cells of wild-type *B. burgdorferi* ML23, which has an available isogenic mutant lacking MnSOD (*sodA*^−^/ΔMnSOD) for comparative studies (67), as grown in liquid BSK-H medium, both with and without Mn supplementation. In this effort, we measured Mn^2+^ speciation, with EPR spectroscopic measurements revealing the partitioning of Mn between H-Mn and L-Mn pools, while ENDOR characterizes the metabolite binding to the H-Mn. The extent of H-Mn antioxidant accumulation as measured by EPR is a strong biological indicator of radiation resistance across the tree of life, including prokaryotes, bacteria and archaea, eukaryotes, including fungi, extremely radiation-resistant nematodes like *Caenorhabditis elegans*, and most recently, whole *Drosophila melanogaster* flies (2, 5, 64). The present study investigates the dynamic relationship between L-Mn proteins and H-Mn antioxidants, along with the relative roles of H-Mn and MnSOD, in providing radioresistance.

We here demonstrate by EPR that *B. burgdorferi* cells dynamically incorporate additional Mn^2+^ in response to MnCl_2_ supplementation, as has been observed for *D. radiodurans* and other free-living organisms, but with one major difference—the spirochetes remain radiosensitive even as they hyperaccumulate H-Mn antioxidants (20). This unvarying radiosensitivity is only partly explained by the spirochete’s highly unusual linear genome architecture, which we suggest contributes to DNA repair malfunction in irradiated *B. burgdorferi* (45), and we address this issue. We further show that *B. burgdorferi* Mn-binding metabolites are outcompeted for Mn^2+^ by its abundant SodA enzymes. Radioresistance is sharply decreased in the ΔMnSOD mutant, but the diminished radioresistance of this mutant is restored to near wild-type levels by simple MnCl_2_ supplementation. However, with supplementation, the mutant abruptly experiences Mn toxicity as the spirochetes grow older and the metabolite pool shrinks, and with it, the population of H-Mn antioxidants.

## RESULTS

Our study employs EPR and ENDOR spectroscopies to track changes in speciation of Mn²^+^ in intact *B. burgdorferi* cells, in particular monitoring substitutionally labile, antioxidant H-Mn complexes and changes in this pool that occur as the spirochetes transition from exponential to stationary phase. We measure the spirochete’s survivability to acute γ-irradiation, which simulates the respiratory burst of O_2_^•−^ deployed as a critical weapon by the host’s innate immune response. During acute exposure to ROS generated by γ-irradiation in BSK-H medium, long-lived O_2_^•−^ radicals generated from dissolved dioxygen (O_2_) represent an unlimited oxidative challenge at the cell surface, and these are countered by MnSOD at the onset of recovery (Text S2). In contrast, the limited O_2_^•−^ radicals that form and are trapped in the cytoplasm are countered by H-Mn metabolite antioxidants. Thus, this pair of defenses—MnSOD and H-Mn—together address the effects of the respiratory superoxide burst deployed by the host’s innate immune response, which is directed at the pathogen’s surface (37).

**Fig 1:**
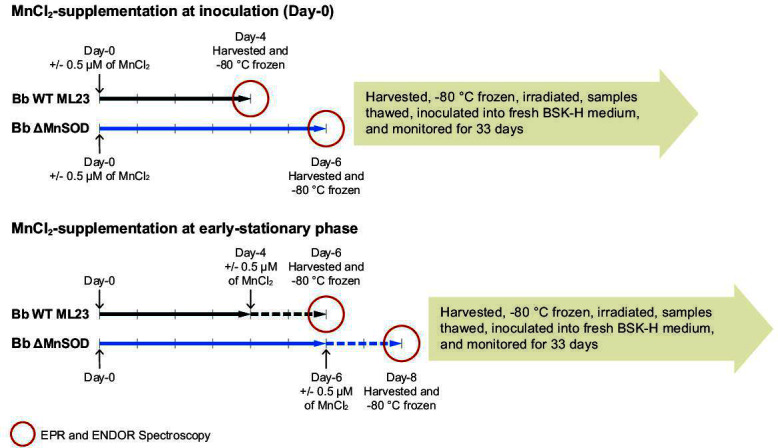
Timelines for alternative protocols of growth, Mn supplementation, irradiation, and recovery, in parallel with spectroscopic investigation for WT *B. burgdorferi* and the ΔMnSOD mutant. As defined in the Introduction, in stationary phase cultures, *B. burgdorferi* experiences nutrient limitation.

H-Mn complexes accumulate in resistant cell types during exponential growth, reaching peak concentrations in early-stationary phase cells, where these cells are the most radiation-resistant when optimally supplemented with MnCl_2_ pre-irradiation (20), and as defined here by achievement of peak cell density (~10⁸ cells/mL). As cells grow older and metabolites in the medium become depleted, the H-Mn antioxidant content decreases, and radiation survivability declines accordingly (5, 22, 53, 56). In testing the corresponding responses to radiation of stationary phase cultures of *B. burgdorferi*, we examined cultures supplemented or not with MnCl_2_ pre-irradiation in log phase, or instead with older (early-stationary phase) spirochetes. Thus, our measurements of survivability conveniently examine the Mn-dependent mechanisms that protect both metabolite-replete (log phase) and metabolite-depleted (stationary phase) spirochetes against O_2_^•−^ generated by radiation, as well as by radiation-damaged metabolic machinery during recovery (45, 68–70). Figure 1 outlines and illustrates the strategies and the two timelines of the study, which are next described in detail.

The MnCl_2_ supplementation of *B. burgdorferi* in the absence of irradiation was performed at two time points: either at the time of inoculation of cells into fresh BSK-H medium, or at a later time when cells had begun to enter the stationary phase, with metabolite-depleted BSK-H medium. The details of our experimental approach are illustrated in Fig. 1: the wild-type *B. burgdorferi* strain ML23 and its isogenic *sodA^−^* disruption mutant ΔMnSOD were grown in BSK-H medium supplemented or not with 0.5 µM MnCl_₂_ upon inoculation, denoted day-0 (metabolite replete) (~10^6^ cells/mL), as indicated in the upper timeline, or they were supplemented at the early-stationary phase day-4/day-6 (metabolite depleted) (~10^8^ cells/mL), as indicated by the lower timeline. Bacteria were harvested and stored at −80°C for spectroscopic experiments and for subsequent examination of the effects of radiation. Red circles mark the time points at which EPR and ENDOR spectroscopies were performed (1, 45, 48, 61–64). To monitor spirochete viability after irradiation, the harvested −80°C frozen samples were irradiated, thawed, and inoculated into fresh medium without (further) supplementation, thereby defining a new day-0 for growth. Spirochetes were visually monitored for changes in cell number and metabolic activity over 33 days using dark field microscopy (45). We sought to further characterize the radiation survival limit of *B. burgdorferi*, defined as that ionizing radiation dose at which inoculated cells do not replicate, or more specifically, the dose (kGy) required to kill ~10^6^ spirochetes when the cells are irradiated frozen and then recovered in standard BSK-H medium (45).

Importantly, radioresistance in *B. burgdorferi* cannot be assessed by colony-forming unit analysis on solid medium because spirochetes proliferate very slowly on agar and exhibit considerable heterogeneity in colony size, and this is why we gauge radiosurvivability post-radiation in liquid culture as the failure of an irradiated culture of 10^6^ *B. burgdorferi* cells to resume growth (45). In the current study of radiosurvivability, WT and ΔMnSOD spirochetes were harvested at a matched physiological state: day 4/6 for WT/ΔMnSOD at early-stationary phase, as defined by achievement of peak cell density (~10⁸ cells/mL)—or at late-stationary phase (day 6/8 for WT/ ΔMnSOD), namely, once the spirochetes stopped replicating. We note that the growth lag of *B. burgdorferi* ΔMnSOD is independent of freezing and mirrors the lag observed in liquid cultures reported for *D. radiodurans sodA*^−^ mutants (5) (Fig. 1).

We examined the role of MnSOD by comparing the gamma radiation survival and Mn speciation for the WT with that for an isogenic *sodA*^−^ disruption mutant (ΔMnSOD), also recovered in BSK medium without MnCl_2_ supplementation.

### Mn^2+^ supplementation of *B. burgdorferi*

**Fig 2:**
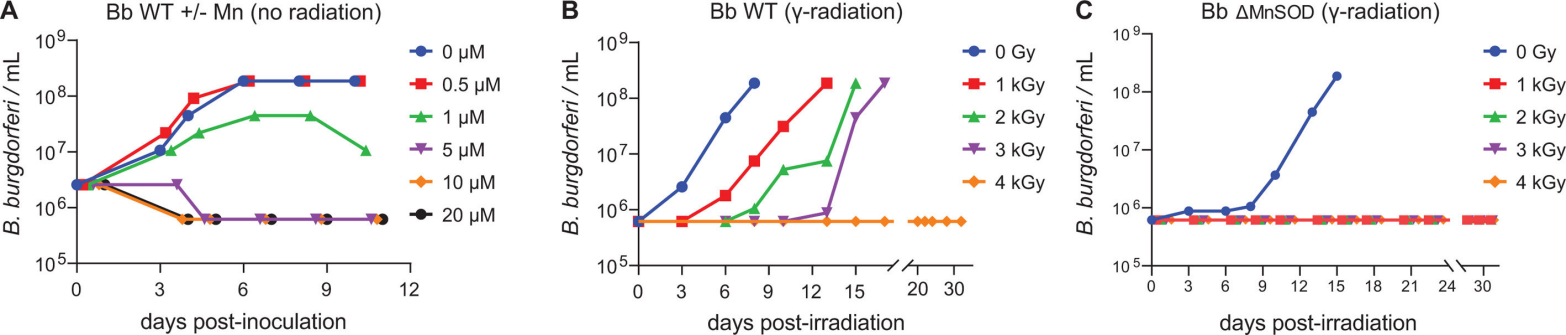
Proliferation and γ-radiation survivability of *B. burgdorferi* (Bb). (**A**) Effect of MnCl_2_ concentration on the growth of WT *B. burgdorferi* (ML23) in BSK-H medium. BSK-H medium was supplemented with MnCl_2_ (0–20 µM) at the time of inoculation. Approximately 10^6^ WT (ML23) spirochetes were inoculated into 10 mL BSK-H medium and visually monitored for proliferative growth and motility by dark-field microscopy for 11 days (45). By definition, exponentially growing cells (day-0; Fig. 1) in BSK-H medium are replete in metabolites, whereas early-stationary phase (~10^8^ cells/mL) cells are increasingly depleted in metabolites as the spirochetes grow older (69). The WT cells reach the early-stationary phase of growth after 4 days in standard BSK-H medium supplemented or not with 0.5 µM MnCl_2_, but not at higher concentrations, which are toxic. (**B**) Gamma radiation survival limit of WT *B. burgdorferi* (ML23), as defined above. Following irradiation (0–4 kGy), ~10^6^ ML23 cells were inoculated into 10 mL BSK-H medium and visually monitored for growth and motility by dark-field microscopy for 33 days. The survival limit of ~10^6^ WT ML23 cells in standard BSK-H is <4 kGy. (**C**) Gamma radiation survival limit of ΔMnSOD *B. burgdorferi* (*sodA*^−^); growth conditions and monitoring as described in panel B. In contrast, the survival limit of ~10^6^ ΔMnSOD (*sodA*^−^) cells in standard BSK-H is <1 kGy.

We first tested the effects of Mn²^+^ supplementation on the growth of non-irradiated WT *B. burgdorferi* by adding MnCl_₂_ at concentrations of 0.5, 1.0, 5.0, 10, and 20 µM to BSK-H medium at the time of inoculation (day 0). This experiment showed that supplementation with 0.5 µM MnCl_₂_ negligibly enhanced the growth of WT *B. burgdorferi* (ML23) under standard conditions. However, supplementation of WT with just 1 µM or higher concentrations was toxic (Fig. 2A). Next, we measured the radiation survival limit of control samples of WT (Fig. 2B) and ΔMnSOD (Fig. 2C) *B. burgdorferi* cells without Mn supplementation. The WT and ΔMnSOD *B. burgdorferi* cells grown in BSK-H medium without Mn-supplementation have a survival limit of <4 and <1 kGy, respectively.

### Mn supplementation at the time of inoculation (day-0) and radiosurvivability

**Fig 3:**
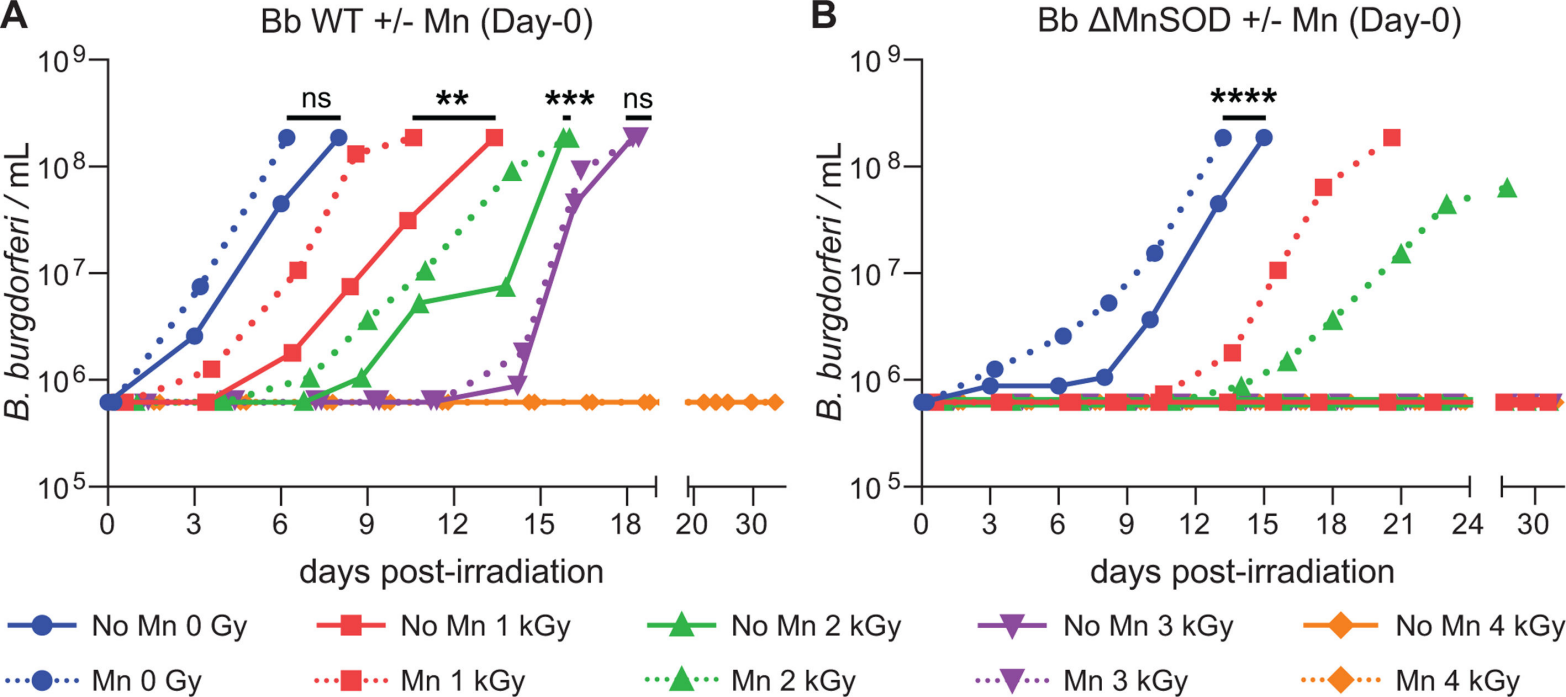
*B. burgdorferi* γ-radiation survival limit in response to Mn supplementation at the time of inoculation (day-0) (see Fig. 1). (**A**) Radiation survivability of Bb WT ML23. Spirochetes were first cultured in BSK-H medium supplemented (Mn) or not (No Mn) on day-0 with 0.5 µM MnCl_2_ and grown to the early-stationary phase (~10^8^ cells/mL), then frozen and irradiated to the indicated dose (0–4 kGy) (Fig. 1). Following irradiation, ~10^6^ cells were inoculated into 10 mL standard BSK-H medium and visually monitored for proliferation and motility by dark-field microscopy for 33 days. When Mn was supplemented at day-0, the survival limit of ~10^6^ WT cells remains unchanged <4 kGy. (**B**) Radiation survivability of Bb ΔMnSOD. *sodA^−^* mutant cultured in BSK-H medium supplemented (Mn) or not (No Mn) on day-0 with 0.5 µM MnCl_2_, grown to the early-stationary phase, then frozen and irradiated to the indicated dose (0–4 kGy) (Fig. 1). Survivability assays as in the legend to panel A. The survival limit of ~10^6^ ΔMnSOD cells is <1 kGy without Mn supplementation and increased to <3 kGy when supplemented with 0.5 µM MnCl_2_ on day-0.

We then measured radiation survival under 0.5 μM Mn supplementation pre-irradiation. As shown in Fig. 3A, the survival limit of irradiated WT remains unchanged (<4 kGy) after 0.5 μM MnCl_2_ supplementation at the time of inoculation of the irradiated cells (day-0; Fig. 1). However, while MnCl_2_ supplementation did not increase the survival limit of the WT, supplementation does accelerate the recovery rate by shortening the growth lag subsequent to a given radiation dose of 1 kGy (*P* = 0.0116) and 2 kGy (*P* = 0.0002); 0 Gy and 3 kGy did not show statistical differences.

In the absence of MnCl_2_ supplementation, the survival limit of ΔMnSOD *B. burgdorferi* cells is only <1 kGy, significantly lower than the survival limit of the WT (<4 kGy). However, Mn supplementation restores the diminished radioresistance of the ΔMnSOD mutant to near WT levels: the survival limit of ΔMnSOD supplemented post-irradiation with 0.5 μM MnCl_2_ at day-0 is increased from <1 kGy to <3 kGy, and MnCl_2_ supplementation led to more rapid growth in the absence of irradiation (*P* < 0.0001) (Fig. 3B). This difference shows that radiation resistance in WT *B. burgdorferi* is provided mainly by MnSOD, but Mn accumulation through Mn supplementation compensates for the loss of MnSOD function in the *sodA*^−^ mutant (Fig. 3B), as well as accelerating the onset of proliferative growth post-irradiation of WT (Fig. 3A).

### Mn supplementation at early-stationary phase and subsequent radiosurvivability

**Fig 4:**
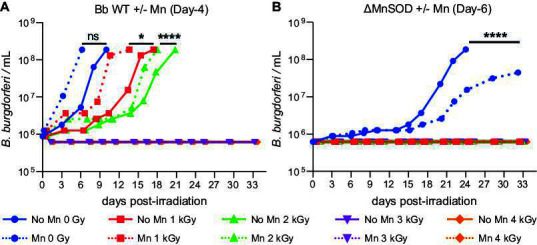
Recovery and survival of *B. burgdorferi* cells post-γ-radiation (survival limit) in response to MnCl_2_ supplementation pre-irradiation, but at the early-stationary phase (see Fig. 1). (**A**) Radiation survivability of Bb WT (ML23). Spirochetes cultured in BSK-H medium supplemented (Mn) or not (no Mn) on day-4 with 0.5 µM MnCl_2_ and harvested 2 days later. The day-4-supplemented cells were frozen and irradiated to the indicated dose (0–4 kGy). Survivability assays as in the legend to Fig. 3. The survival limit of ~10^6^ WT cells supplemented on day-4 is <3 kGy with Mn supplementation. (**B**) Enhanced Mn toxicity in Bb ΔMnSOD. *sodA*^−^ cultured in BSK-H medium supplemented (Mn) or not (no Mn) on day-6 with 0.5 µM MnCl_2_ and harvested 2 days later. The day-6-supplemented cells were frozen and irradiated to the indicated dose (0–4 kGy). Survivability assays as in the legend to Fig. 3. The survival limit of ~10^6^ ΔMnSOD cells supplemented on day-6 is <1 kGy with or without Mn supplementation; however, abrupt Mn toxicity is evident in non-irradiated ΔMnSOD cells upon Mn supplementation on day-6.

We next studied the effect of MnCl_2_ supplementation on the growth and radiation survivability of both WT and ΔMnSOD *B. burgdorferi* when 0.5 μM MnCl_2_ was added at an early-stationary phase of growth of unirradiated cells: on day-4 for WT; day-6 for the ΔMnSOD mutant (Fig. 1). Subsequent to such Mn supplementation, the cells were incubated for an additional 2 days to equilibrate. They were then frozen, irradiated, and finally recovered in standard BSK-H for 33 days. Cell proliferation and motility of spirochetes within the recovering culture were monitored by dark-field microscopy for 33 days (Fig. 4A and B).

The survival data reveal that when supplemented with 0.5 µM MnCl_₂_ on either day-0 or day-4, WT survival behavior remained essentially unchanged over the 33-day time course, although the survival limit of WT cells supplemented on day-4 was reduced from <4 kGy to <3 kGy, and the statistical differences remained in 1 kGy (*P* = 0.0370) and 2 kGy (*P* < 0.0001) Fig. 4A). In contrast, the opposite trend was observed in the ΔMnSOD mutant—Mn supplementation on day-6 did not improve recovery, and acute Mn toxicity was evident even in the non-irradiated ΔMnSOD control when supplemented on day-6 (*P* < 0.0001) (Fig. 4B). This Mn toxicity is attributed to off-target binding effects (see below).

### Assessing H-Mn antioxidant content of *B. burgdorferi* through EPR

Our recent EPR study of *B. burgdorferi* strain B31 revealed a low H-Mn population in WT cells, correlating with the marked radiosensitivity (45). In the present study, we examined *B. burgdorferi* strain ML23 in greater detail. ML23 is closely related to B31 but offers the advantage of the availability of its isogenic *sodA*-deficient (ΔMnSOD) mutant for comparative analysis (67).

**Fig 5:**
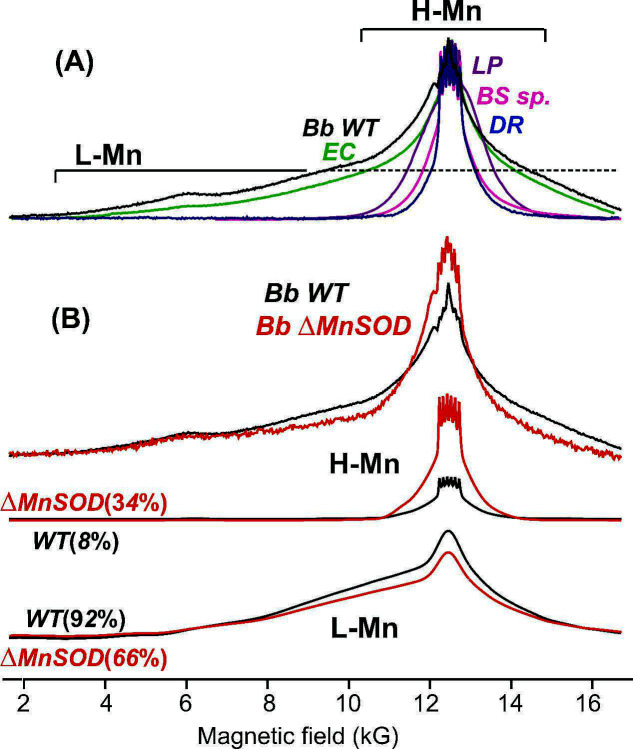
35 GHz Pulse EPR spectra. (**A**) Overlaid spectra, scaled to the same maximum amplitude at ~12 kG, of *B. burgdorferi* (ML23 *Bb* WT cells, black) and radiation-sensitive *E. coli* (*EC*; green); as indicated, these spectra are broad because they contain a dominant L-Mn pool. Also overlaid are spectra of radioresistant *D. radiodurans* (*DR*; blue), *L. plantarum* (*LP*; purple), and *B. subtilis* spores (*BS*; magenta), with spectra dominated by narrow signals from H-Mn antioxidants and negligible contribution from L-Mn. (**B**, upper) ML23 cells WT (black) and ΔMnSOD (*sodA*^−^) (red) grown in standard BSK-H medium without Mn supplementation. (**B**, middle) Partitioning of Mn^2+^ EPR spectra of WT and *sodA^−^* cells into contributions from H-Mn and (**B**, lower) L-Mn (see Fig. S1 for details). The strains were grown to the early-stationary phase prior to EPR. Experimental conditions as in Materials and Methods (2, 5, 45).

As controls for the effects of Mn supplementation at the time of inoculation, EPR experiments (Fig. 5) were first conducted on intact, non-irradiated WT and ΔMnSOD mutant spirochetes grown in standard BSK-H medium without Mn supplementation, with WT harvested on day-4, and mutant (*sodA*^−^) harvested on day-6 (Fig. 1 and 2A).

The 35 GHz, pulsed EPR (absorption display) spectrum of cellular manganese is characteristic of high spin (*S* = 5/2) Mn^2+^ and can be partitioned into two Mn pools, each identified by its distinctive EPR spectrum (2). One pool displays the EPR spectrum of high-symmetry Mn^2+^ complexes with a narrow magnetic field span and with six sharp peaks at its center due to ^55^Mn electron-nuclear hyperfine interaction (labeled as H-Mn). This pool includes non-enzymatic complexes with Mn-metabolites of low-molecular weight as ligands, including orthophosphate (Pi), carboxylates, peptides, carboxylic acids, and amino acids (15). The second Mn-pool displays a broad featureless EPR spectrum that extends from very low field to beyond the available spectrometer magnetic field. This pool, denoted L-Mn, contains a heterogeneous population of low-symmetry, tightly chelated Mn complexes, largely Mn enzymes, including the antioxidant enzyme MnSOD (2, 48).

In the *B. burgdorferi* WT grown in standard BSK-H medium without additional MnCl_2_ supplementation and harvested at the early-stationary phase, Mn is mostly present as the L-Mn pool (~90%), with only a small H-Mn pool (~10%), as shown by the decomposition of the Mn-EPR spectra (Fig. 5A and B; Fig. S1). With this low H-Mn population, the *B. burgdorferi* are similar to the radiation-sensitive *E. coli* (45). In contrast, radiation-resistant free-living bacteria (*D. radiodurans, B. subtilis*, and *L. plantarum*) typically show a narrow spectrum arising from a very high percentage of the pool of H-Mn (>90%) (Fig. 5A).

A strong correlation between fraction of H-Mn and radiation resistance (2) predicts that *B. burgdorferi,* with its low fraction of H-Mn, should be radiation sensitive (Fig. 5B), and that indeed is true as shown by the radiation-survival measurements (Fig. 3 and 4) (45). The EPR spectrum of ΔMnSOD (Fig. 5B, top) shows a modest loss of L-Mn that can be attributed to loss of SOD from WT, but the H-Mn fraction (34%) is still low compared to that of radiation-resistant free-living bacteria (>90%; DR, BS, and LP) that accumulate similar total amounts of cellular Mn (20, 27, 71, 72). Thus, the EPR-derived Mn speciation predicts that both WT and ΔMnSOD *B. burgdorferi*, with low H-Mn population, would be radiation sensitive when grown without Mn supplementation in standard BSK-H medium, as observed, and that MnSOD thus is the primary defense against ROS.

### Mn supplementation and Mn speciation by EPR

We next conducted EPR measurements of non-irradiated WT and ΔMnSOD *B. burgdorferi* grown in BSK-H medium supplemented with 0.5 μM MnCl_2_ on day-0 and examined by EPR on day-4, or supplemented at early-stationary phase (day-4/6) for WT/mutant. The day-0 and day-4/6 cells, respectively, represent nutrient-replete cells (exponential growth) and nutrient-depleted cells (early-stationary phase) (Fig. 1, 3, and 4). Cells Mn-supplemented on day-4/6 were collected for EPR 2 days after supplementation to allow Mn to equilibrate in cells. These measurements were compared to those of the corresponding control samples without Mn supplementation.

**Fig 6:**
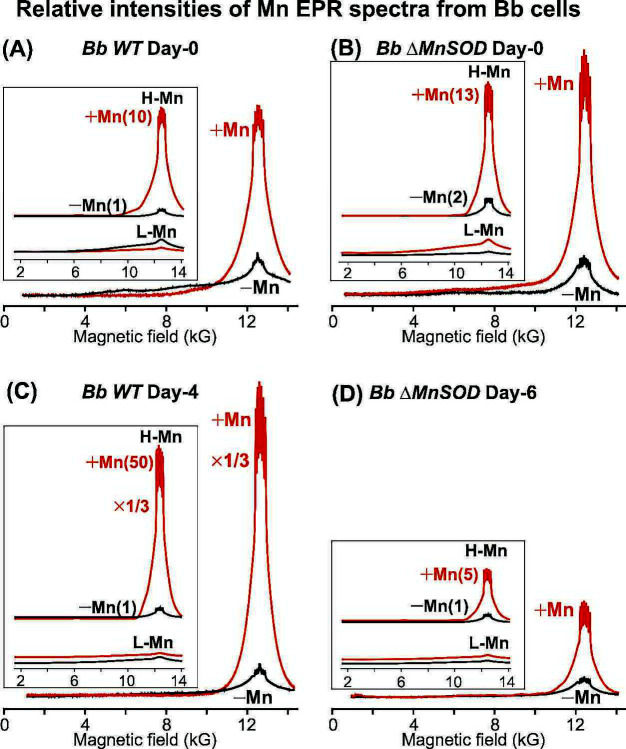
Relative intensities of Mn EPR spectra from *B. burgdorferi* cells. Continuous-wave absorption-display EPR spectra (2 K, 35 GHz) listing relative EPR intensity of non-irradiated *B. burgdorferi* (*Bb*) spirochetes of Fig. 3 and 4 before (−Mn black) and after (+Mn red) supplementation with 0.5 μM MnCl_2_. (**A**) WT and (**B**) *sodA*^−^ day-0 Mn supplementation at the time of inoculation. (**C**) WT early-stationary phase Mn day-4 supplementation (early-stationary phase cells). The actual + Mn EPR spectrum height is divided by three. (**D**) *sodA*^−^ early-stationary phase Mn supplementation on day-6. Insets show partitioning of EPR spectra into contributions from H-Mn and L-Mn (see Fig. S2 through S5 for details). The relative H-Mn contributions are scaled by assigning the lowest H-Mn intensity as 1. For experimental conditions, see Materials and Methods.

Upon 0.5 μM MnCl_2_ supplementation in the BSK-H media at the time of inoculation of growth (day-0), both WT and ΔMnSOD *B. burgdorferi* cells hyperaccumulate manganese (Fig. 6A and B). Importantly, decomposition of the spectra into contributions of H-Mn and L-Mn (inset, Fig. 6A and B; Fig. S2 and S3) confirms that almost all cellular Mn acquired upon supplementation is present as H-Mn, with minimal increase of L-Mn, consistent with the model that Mn accumulates in cells as H-Mn only after Mn-dependent proteins, including SodA, are fully metalated, as detailed previously (5, 10). The accumulated H-Mn of wild-type Mn-supplemented *Borrelia* is 10-fold greater than the H-Mn of WT cells grown without Mn supplementation (inset, Fig. 6A). Mn supplementation of ΔMnSOD causes a slightly greater increase in H-Mn (~×13) than WT (~×10), leading to an approximately sevenfold more H-Mn than ΔMnSOD without supplementation (inset, Fig. 6B). This substitutionally labile pool of Mn-metabolite complexes (H-Mn) generated by Mn supplementation in *B. burgdorferi* is at similar levels to that of H-Mn present in radiation-resistant free-living bacteria *D. radiodurans*, *L. plantarum*, and *B. subtilis* (Fig. 5A) (20, 65, 73).

EPR results thus suggest that Mn-supplemented (+Mn) cells, both WT and ΔMnSOD *B. burgdorferi*, should show enhanced radiation resistance. In fact, both WT and ΔMnSOD show higher growth rates with Mn supplementation post-irradiation (Fig. 3), as noted above, but the radiation survival limit for WT nonetheless remains essentially unchanged post-MnCl_2_ supplementation (+Mn) (perhaps even slightly lowered, from <4 Gy to <3 kGy). However, the increased pool of H-Mn antioxidants for ΔMnSOD roughly triples radiosurvivability (increase from <1 kGy to <3 kGy) (Fig. 3), making it comparable to that of WT. Thus, H-Mn antioxidants must be functioning as a complement to MnSOD for protection against O_2_^•−^ as its absence lowers the radiosurvivability limit, which is restored by increasing H-Mn.

It is nonetheless puzzling that the radiation survival limit for WT remains unchanged upon Mn supplementation (+Mn <4 kGy), even though it strongly accumulates H-Mn post-Mn supplementation. As discussed below, we attribute this to the following two causes that are predicted to hinder DNA DSB repair: (i) the spirochetes lack the DNA damage SOS response, a mechanism that facilitates DSB repair and helps cells survive radiation, and instead rely on nucleotide excision repair (74, 75), and (ii) the cells’ linear genome configuration (45).

We further tracked by EPR the Mn speciation of *B. burgdorferi* WT/mutant cells when Mn was supplemented at the early-stationary phase (Fig. 1, 6C, and D). As noted above and further discussed below, MnSOD must protect the surface proteins of *B. burgdorferi* from ROS, including its Mn transporters—so in the absence of MnSOD, post-irradiation cells would be expected not only to grow more slowly than the WT, but the mutant would be expected to accumulate less H-Mn in the stationary phase because the cells rely entirely on transporters for essential nutrients (5, 41, 43). Consistent with this, WT *B. burgdorferi* grows significantly faster than ΔMnSOD and shows the highest increase (50×) in the uptake of Mn and H-Mn upon day-4 supplementation (inset, Fig. 6C), whereas the ΔMnSOD mutant shows toxicity with Mn supplementation at 0.5 µM in early-stationary phase cells (day-6), with only 5× increase in Mn compared to the 50× increase for WT (compare Fig. 6C and D).

### Assessing H-Mn ligation

The findings above address not only the benefit of H-Mn accumulation, both as providing a potential reservoir of Mn^2+^ ions for MnSOD in *B. burgdorferi* and for radioresistance (Fig. 3) but also highlight the danger of Mn overaccumulation (Mn toxicity) observed in the ΔMnSOD mutant as it grows older (Fig. 4). ENDOR spectroscopy probes the H-Mn speciation/ligation, which reflects the composition of the Mn-metabolite pool. Since hyperaccumulated Mn is stored as H-Mn in all Mn-supplemented samples, we performed ENDOR spectroscopy on the supplemented WT/ΔMnSOD *B. burgdorferi* day-0/day-4, day-6 to track Mn-metabolite ligation of the accumulated H-Mn pool at exponential and stationary states, thereby determining whether the composition of the Mn-metabolite pool is altered as part of the Mn-toxicity in ΔMnSOD cells when supplemented at the early-stationary phase (Fig. 1).

**Fig 7:**
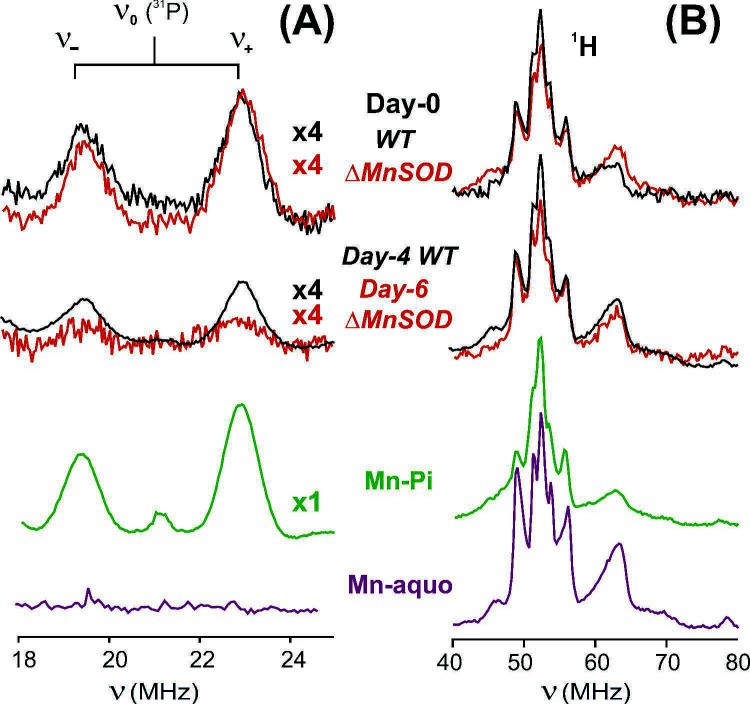
Pulsed 35 GHz, 2 K (**A**) ^31^P ReMims ENDOR and (**B**) ^1^H Davies ENDOR spectra of Mn-supplemented *B. burgdorferi* WT/ΔMnSOD for day-0 and day-4/6 time points (see the text). ENDOR spectra are normalized to the Echo height, so ^1^H, ^31^P ENDOR peak amplitudes scale with average Mn-Pi speciation. ×4, amplitude of spectrum is magnified four times. For conditions, see Materials and Methods.

Figure 7 shows 2 K, 35 GHz ^1^H Davies ENDOR and ^31^P refocused Mims (ReMims) ENDOR spectra of WT/ΔMnSOD *B. burgdorferi* supplemented with 0.5 μM MnCl_2_ on day-0 or day-4/6 (Fig. 1). ^14^N electron spin echo envelope modulation (ESEEM) spectra show an absence of bound nitrogenous ligands (Fig. S6). The ^1^H ENDOR spectra from the H-Mn pool in all *B. burgdorferi* samples show patterns with peaks of lower amplitude than for Mn-aquo, indicating that bound metabolites replace bound H_2_O. The ^1^H spectra of all *B. burgdorferi* samples are of essentially equal intensity, which means that the number of Mn-bound H_2_O is very similar in all samples, and thus the total fraction of Mn^2+^ with either a bound Pi or a bound ENDOR-silent metabolite is the same. The observation of variations in the fraction of Mn-Pi, but unvarying amounts of bound waters, indicates that there is a varying fraction of bound ENDOR-silent metabolites, primarily carboxylates. This is as expected because the ligation of a solution Mn^2+^ is controlled by the metabolite pool in which it is located. *B. burgdorferi* cells are entirely dependent on the host (or BSK-H medium) for primary metabolites, thus their metabolite pool reflects that of the host (or the medium) population (76); this contrasts with metabolically proficient free-living bacteria like *D. radiodurans*, which not only express myriad metabolite transporters but, critically, can also synthesize their own pool of metabolites, which changes in response to irradiation (41, 77).

The ^31^P ENDOR spectra of *B. burgdorferi* WT and ΔMnSOD cells supplemented with MnCl_2_ on day-0 both show ~1/4 the intensity of that of Mn-Pi (Fig. 7A), which means that ~25% of the cellular H-Mn binds a Pi, and that the fraction of Mn-Pi is the same for both WT and ΔMnSOD cells in the same growth medium. Thus, it is the increased amount of H-Mn antioxidants in supplemented ΔMnSOD, not a change in ligation, that roughly triples radiosurvivability, a level of radioresistance (increase from <1 kGy to <3 kGy) (Fig. 3), making it comparable to that of WT, with H-Mn antioxidants replacing MnSOD for protection against O_2_^•−^.

With day-4 supplementation (monitored 2 days subsequently), the Pi ENDOR response for WT is reduced to ~1/2 compared to that for day-0, but day-4-supplemented *B. burgdorferi* compensates for this decrease in the fractional population of Mn-Pi by accumulating five times more H-Mn (day-4, 50×) than day-0 (10×) supplemented WT cells (Fig. 6C versus Fig. 6A). Overall, this leads to similar or higher levels of Mn-Pi for WT cells as in day-0, with similar radiosurvival of WT cells whether Mn was supplemented at day-0 or day-4 (Fig. 4A). However, the day-6 Mn-supplemented ΔMnSOD cells exhibit a very low level of H-Mn (5× lower, Fig. 6D), while a further decrease in the Mn-Pi component of that pool further indicates an extensive depletion of Pi (presumably with other metabolites) in cultures transitioning from log phase to stationary phase. We note that BSK-H medium is supplemented with sodium phosphate monobasic (0.115 g/L; 1 mM) (Materials and Methods). Thus, the differences in Pi concentrations indicated by the differing extent of Pi ligation in the H-Mn show that they are not controlled by equilibration with the medium, with the cellular Pi concentration being decreased by a decrease in the efficiency of the spirochetes’ transport systems post-irradiation (see below). Overall, the Pi pool, whose concentration determines the percentage of Mn with bound Pi, which in turn dominates the effectiveness of the H-Mn as an antioxidant, is similar in log phase and early-stationary phase WT cells, but diminished in ΔMnSOD cells (Fig. 6D), which we attribute to radiation damage of Pi and Mn surface transporters.

## DISCUSSION

The ability of manganese to scavenge superoxide (O_2_^•−^) radicals is vital to all aerobic biota, in particular, providing resistance to radiation and desiccation, and contributing to longevity and pathogenicity of microorganisms (4, 29). Mn^2+^ prevents O_2_^•−^ poisoning both as a metal cofactor in the ubiquitous enzyme MnSOD, as first reported by Fridovich and colleagues (11, 12, 78, 79), and as a catalytic center in small-molecule Mn antioxidants (H-Mn) (2, 4, 5, 18, 65). Our recent findings (5) support the idea that global responses to oxidative stress must be understood through an extended theory that includes the H-Mn antioxidants as potent O_2_^•−^ scavengers that complement, and can even supplant, MnSOD. Our current work tested this extended theory on a Mn-dependent bacterial pathogen, *B. burgdorferi* (WT-ML23 and ΔMnSOD, its isogenic mutant), by studying the effects of MnCl_2_ supplementation on the spirochete’s growth and radiation survivability (Fig. 2 through 4).

As part of expanding therapeutic strategies against Lyme disease, now including approaches to vaccine development that combine H-Mn antioxidants with gamma radiation sterilization (18, 45, 66, 80), we applied EPR and ENDOR spectroscopies to deepen our understanding of the Mn-dependent oxidative stress defenses of *B. burgdorferi* (Fig. 5 through 7). We compared *B. burgdorferi* to *D. radiodurans*, *L. plantarum*, and *B. subtilis*—innocuous, widespread, and highly radiation-resistant free-living bacteria with well-characterized Mn-dependent antioxidant defenses that strategically utilize MnSOD and H-Mn antioxidants (2, 5, 20, 25, 65, 73, 81, 82). These free-living bacteria, which have similar capacities to accumulate Mn atoms as *B. burgdorferi* (83, 84) on a per-cell basis (20), provide valuable comparisons for studying Mn homeostasis in pathogens (33, 85).

The inability of O_2_^•−^ to cross lipid membranes due to its negative charge is central to understanding the complementary roles and locations of MnSOD and Mn antioxidants (38). Extreme oxidative stress resistance in free-living bacteria is best explained by an extended model in which (i) MnSOD is the primary protectant of surface and periplasmic proteins directly exposed to atmospheric O_2_ during desiccation, which yields O_2_^•−^ via the release of adventitious electrons from damaged respiratory enzymes (5, 15, 21, 68, 86); and (ii) H-Mn antioxidants, located in the cytoplasm, protect proteins involved in transcription, translation, and DNA repair of DSBs caused by O_2_^•−^ generated during irradiation (22) and damaged respiratory systems. Because desiccation first impacts the cell’s outer surfaces, including the periplasm, respiratory chain proteins are likely early targets of oxidative damage (87). Converging lines of indirect evidence support this: H-Mn antioxidants, which scavenge O_2_^•−^ but not H_₂_O_₂_, confer extreme desiccation resistance on purified enzymes simply dried under atmospheric O_₂_ (15); however, in *D. radiodurans*, H-Mn is cytoplasmic (22), leaving the cell surface vulnerable to O_2_^•−^ attack during drying or exposure to extracellular O_2_^•−^ generators such as paraquat. Consistently, *sodA^−^* deletion mutants of *D. radiodurans* are hypersensitive to desiccation and paraquat, yet retain WT levels of radioresistance. We therefore conclude that respiratory systems of aerobic microorganisms like *D. radiodurans* require MnSOD in surface-proximal environments for protection against O_2_^•−^ during desiccation (5, 19).

Importantly, pathogenic bacteria are subject to copious extracellular O_2_^•−^ produced by a host’s innate immune system, whereas acute irradiation generates both extracellular and intracellular O_2_^•−^ radicals (23, 35, 37); the latter can also be produced by damaged energy-producing pathways during recovery (43). Thus, exposure of pathogens to acute γ-irradiation is a comprehensive technique, able to probe oxidative-defense against both extracellular and intracellular O_2_^•−^ radicals, and in doing so provides a means to separate the antioxidant role of MnSOD and H-Mn.

Superoxide radicals generated either by the host’s ROS-based immune mechanisms or through radiolysis preferentially oxidize proteins (19, 43, 66). As O_2_^•−^ is a charged species at physiological pH (p*K*a = 4.8) and does not easily cross membranes (38), the host’s innate immune system naturally targets *Borrelia’s* surface, inactivating transporters vital to the spirochete’s metabolite acquisition (34, 76, 88), and defense against this attack is provided by MnSOD (Text S1 and S2).

**Fig 8:**
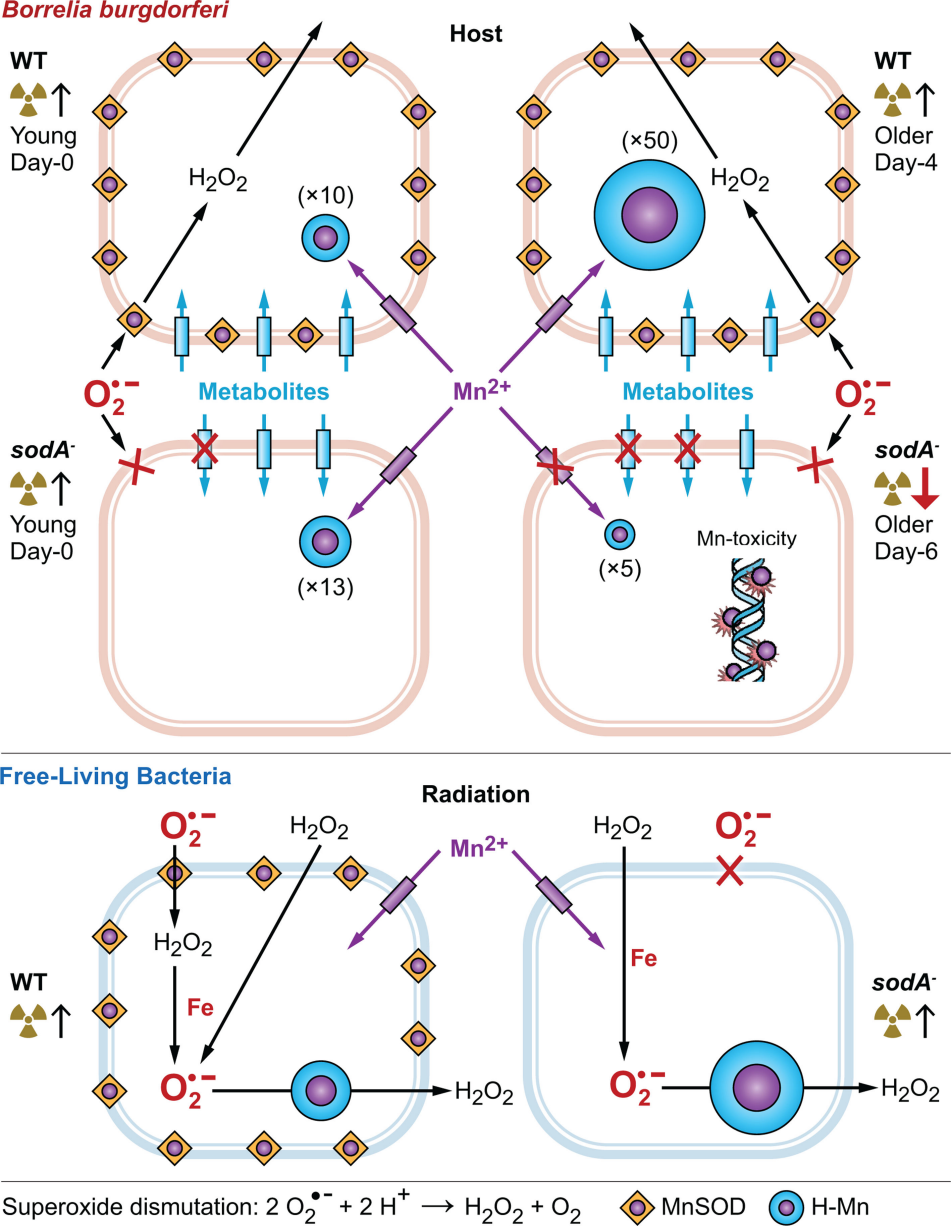
*Borrelia burgdorferi* strategically metalates MnSOD and Mn-metabolite complexes (H-Mn) to counteract superoxide (O_2_^•−^) damage. This model illustrates the equilibrium between Mn²^+^ ions (purple) bound in MnSOD enzymes (orange square) at the cell periphery and Mn²^+^ bound in the cytoplasmic H-Mn pool (turquoise circle) in *B. burgdorferi* supplemented with MnCl_2_ (top), compared to free-living bacteria (e.g., *D. radiodurans*) (bottom) (19). Consistent with the absence of Fe-based redox ROS chemistry in *B. burgdorferi*, the spirochete does not encode any catalases and peroxidases (91), which are ubiquitous among free-living Mn bacteria (92, 93). Importantly, we infer that MnSOD in *B. burgdorferi* protects the cell boundary from O_2_^•−^ damage, including the periplasm and outer membrane proteins, where the inner membrane forms the final barrier to O_2_^•−^ entering the cytoplasm (Text S1). We infer that H-Mn accumulated in the spirochetes serves mainly as a reservoir of substitutionally labile Mn²^+^ to metalate MnSOD, which protects the myriad metabolite transporters (turquoise rectangle) and other proteins at the cell surface (43, 44). Shown inside a *B. burgdorferi* cell in parentheses (×5–×50) is the increase in cellular H-Mn content upon 0.5 µM MnCl_2_-supplementation, as gauged by EPR (Fig. 6); shown outside a cell (trefoil↑↓), the change in radiation survivability (Fig. 3 and 4). Gauged by ENDOR, the H-Mn metabolite pool is highly depleted in stationary phase ΔMnSOD (*sodA^−^*) day-6 cells (Fig. 1), wherein surplus labile Mn²^+^ ions lead to Mn toxicity (Fig. 4 and 7). Because the extremely auxotrophic *B. burgdorferi* would not likely dedicate a significant fraction of its MnSOD inventory to protecting its Fe-free cytoplasm, we assign MnSOD to the periplasm.

Cytoplasmically generated O_2_^•−^ does not target the negatively charged DNA or RNA. Instead, O_2_^•−^ radicals primarily target solvent-exposed [4Fe-4S] groups in enzymes (89). In Mn-bacteria like *Deinococcus*, H-Mn antioxidants protect Fe proteins (both native and heterologous) from O_2_^•−^ under conditions where H_₂_O_₂_ enters the cell from the extracellular environment (20, 22, 41, 42, 90). This H_₂_O_₂_, which can be generated directly by radiolysis or indirectly via the dismutation of O_2_^•−^ by MnSOD at the cell surface during stress (5), can generate O_2_^•−^ by Fe-dependent Haber-Weiss and Fenton reactions. By contrast, in *Borrelia*, H_₂_O_₂_ can safely diffuse out of cells because these pathogens lack Fe, thereby avoiding those oxidative reactions (23) (Fig. 8).

### MnCl_2_ supplementation and radiation survivability

Since *B. burgdorferi* is heavily reliant on Mn for its antioxidant defense, we carried out Mn accumulation by MnCl_2_ supplementation and measured the effects on Mn speciation and radiation survivability. We utilize EPR spectroscopy to probe how the pathogen allocates and utilizes its surplus manganese upon Mn supplementation, examining the partitioning of Mn^2+^ between L-Mn, which consists mostly of Mn enzymes, including MnSOD, and which display broad featureless EPR spectra, and H-Mn, low-molecular-weight Mn-metabolite complexes (<1 kDa) that display narrow EPR spectra (2). We then contrasted wild-type and ΔMnSOD *B. burgdorferi* radiation responses to MnCl_2_ supplementation with the responses to MnCl_2_ in *D. radiodurans*, *L. plantarum, B. subtilis,* and *E. coli* (11, 12, 15, 20, 73, 81).

MnCl_2_ supplementation in bacteria and fungi, and even in nematodes, flies, and mice, typically results in Mn accumulation, usually with enhanced radiation survivability (15, 20, 64, 94–97). Consistent with this trend, we demonstrate that supplementation of *B. burgdorferi* with 0.5 µM MnCl_2_ accelerates the recovery of irradiated *B. burgdorferi*, significantly shortening the growth lag induced by a given radiation dose (Fig. 3).

Our measurements of survivability during post-irradiation recovery characterize the Mn-dependent mechanisms that protect both log phase (metabolite-replete) and early-stationary phase (metabolite-depleted) spirochetes mainly against O_2_^•−^ produced during irradiation (Fig. 3 and 4) (Text S2). Importantly, and unlike most other Mn-dependent organisms, MnCl_2_ supplementation of WT *B. burgdorferi* pre-irradiation does not increase the radiation survival limit of the cells (Fig. 3); the spirochetes recover faster from irradiation, but they do not survive greater doses of gamma rays. *Instead*, the radiation response of *B. burgdorferi* more closely resembles the response of irradiated *B. subtilis*, which also defies the general correlation between increasing H-Mn content and increasing radiation resistance—radiation resistance in these *Bacillus* cells and endospores is independent of Mn content, although Mn content nonetheless is an important factor in determining survivability to other oxidative stressors (73). This apparent contradiction for monogenomic bacteria like *B. subtilis* is readily explained by the fact that they lack genomic redundancy, which is required for efficient homologous repair of DNA broken by radiation, whereas Mn-accumulating polyploid bacteria, like *D. radiodurans*, typically display greatly increased radiation survival limits upon MnCl_2_ supplementation (19, 20, 65, 73).

### Why is *B. burgdorferi* so radiosensitive?

Our findings reveal that Mn accumulation exerts dual, context-dependent effects. In *B. burgdorferi*, MnCl_₂_ supplementation drives H-Mn hyperaccumulation, enhancing the radioresistance of an isogenic Δ*sodA* mutant to WT levels. However, in WT strain ML23, increased H-Mn does not extend the radiation survival limit, which plateaus at ~4 kGy. This contrasts sharply with free-living Mn-dependent bacteria—including radiosensitive *E. coli*—where simple Mn²^+^ supplementation often produces large increases in radiation survival (15, 20). The simplest explanation is that *B. burgdorferi*’s unusual genome architecture—linear chromosomes—fundamentally limits its capacity to survive high-dose radiation. Linear chromosomes are rare among bacteria, with *B. burgdorferi* being the principal exemplar.

Ionizing radiation-induced DSBs caused by radiolytic ^•^OH radicals present the greatest DNA repair challenge because breakage of both strands yields linear DNA fragments that become progressively shorter and more difficult to reassemble as dose increases. Irradiated bacteria with linear genomes are at a disadvantage compared to bacteria with circular chromosomes, which naturally produce fewer DSB fragments (45). Moreover, *B. burgdorferi* appears not to have evolved robust DSB repair pathways (74, 75). This inherent genomic structural limitation means that even large increases in H-Mn content cannot proportionally increase radiation resistance. In our study, WT *B. burgdorferi* supplemented with 0.5 µM MnCl_₂_ increased H-Mn content by 5- to 50-fold, yet the survival limit remained unchanged at <4 kGy (Fig. 3 and 6). Analogously, DNA repair mutants of *D. radiodurans* remain highly radiosensitive despite hyperaccumulating H-Mn (19, 65).

### Mn-binding metabolites are outcompeted for Mn^2+^ by SodA enzymes in *B. burgdorferi*

We consider the effects of loss of the enzyme MnSOD in *B. burgdorferi* within the extended theoretical framework of oxidative defense, which includes competition for Mn between Mn antioxidants and apo-MnSOD. Consistently, we show by EPR that MnSOD loss in *B. burgdorferi* increases the H-Mn/L-Mn ratio of the spirochetes, with gain in H-Mn content comparable to the loss in the L-Mn pool (Fig. 5B and 6). These findings are explained by a competition for Mn^2+^ between MnSOD and Mn metabolites in *B. burgdorferi,* wherein H-Mn, with its metabolite-driven pool of Mn-antioxidants, can only accumulate in cells once holo-SodA enzymes are fully metalated (Figure 6; [5]).

### Variations in speciation of manganese-metabolite complexes

A particular synergism of metabolite complexes of Mn and MnSOD exists in deinococci, where MnSOD protects membrane proteins critical for metabolite import against O_2_^•−^, and thus is required for the maintenance of H-Mn, with its key role in radiation survival (5, 41, 56). Hence, we probed the composition of the H-Mn pool by ENDOR in cultures of *B. burgdorferi* cells as they grow older, supplemented or not with MnCl_2_ (Fig. 7). Significantly, orthophosphate (Pi) is one of the most common metabolites accumulated in free-living bacteria, reaching 25 mM in deinococci, where it serves as a coordinating buffer required to integrate metabolism, biosynthesis, and genome maintenance (15, 41, 56). It also coordinates to the pool of labile Mn^2+^ complexes in the deinococci as the classic H-Mn antioxidant (2). Importantly, the extent of the coordination of Mn^2+^ by Pi in intact cells is readily discerned and quantified by ENDOR, providing insight into the impact of alternating periods of starvation and surfeit on the ebb and flow of H-Mn metabolites in viable cells at exponential and stationary states (1, 5).

Our EPR/ENDOR, combined with radiation survivability studies on *B. burgdorferi*, shows that H-Mn antioxidants complement MnSOD in the spirochetes for protection against O_2_^•−^. As predicted, MnSOD loss increases the pool of H-Mn in *B. burgdorferi* (5), although this pool remains relatively small compared to that of radiation-resistant free-living bacteria. The ENDOR responses reveal that the population and composition of Mn-binding ligands of H-Mn antioxidants are the same in wild-type and ΔMnSOD *B. burgdorferi* cells. In contrast, ENDOR shows that the speciation within the H-Mn pool changes significantly as the spirochetes transition from exponential to stationary state; notably, the Mn-Pi component of the H-Mn diminishes in the early-stationary phase cells, and it is further diminished for ΔMnSOD cells compared to the wild type (Fig. 6D). This offers yet another element, beyond the impact of its linear genome architecture and limited DNA repair machinery, as to why even upon Mn supplementation, the small accumulation (relative to WT) of H-Mn in early-stationary phase ΔMnSOD cells does not protect the cells: the H-Mn observed in ENDOR exhibits minimal ligation by Pi, which is important in conferring antioxidant properties (18, 48).

More broadly, the paramagnetic resonance spectroscopies suggest that the speciation within the pools of bacterial H-Mn antioxidant complexes is species-specific, shaped by each organism’s *finely tuned* metabolism, which controls the mixtures of small-molecule metabolites that form the foundation of the antioxidant H-Mn system across the tree of life (2, 5, 18, 41, 65, 98). In the case of *B. burgdorferi*, the spirochetes encode only a highly restricted metabolism and are therefore extremely dependent on their hosts (or BSK-H medium) for primary metabolites (69, 76, 88). Thus, *B. burgdorferi* does not encode the Fe-dependent TCA cycle, oxidative phosphorylation, or any other Fe-dependent pathways for *de novo* biosynthesis of amino acids, carbohydrates, or lipids. The spirochete, instead, encodes an array of metabolite transporters that support glycolysis in the presence of O_2_ for ATP production, yielding lactate as the major accumulated end product of energy metabolism (33, 34, 59, 70, 88).

Notably, lactate (C_3_H_5_O_3_⁻) co-accumulates with H-Mn in radioresistant *L. plantarum* (Fig. 5) and in cultured human cancer cells exhibiting the Warburg effect under high-glucose conditions (2, 99–101). In *B. burgdorferi*, the lactate pool likely contributes to the ligation within the H-Mn pool, consistent with the ENDOR data that show that H-Mn is extensively ligated by oxygen-bound carbon-based ligands, namely carboxylates (Fig. 7 and 8). Beyond that, in stationary phase cultures, *B. burgdorferi* experiences not only starvation but also gradual acidification of its environment, a consequence of glucose fermentation to lactate (69). This is further corroborated by ENDOR data, which show an increase in ENDOR-silent ligands to H-Mn, presumably the carboxylates, in stationary phase cultures relative to what is found in the exponential phase (Fig. 7).

### Mn toxicity

Manganese accumulation is beneficial—until it is not. Strong, favorable effects on growth with increasing trace levels of Mn^2+^ ions have been reported for exponential phase cells of *D. radiodurans*, *L. plantarum*, and *B. subtilis*, but not for older, stationary phase cells. In exponential *D. radiodurans*, supplementation with MnCl_2_ from 5 nM to 5 µM greatly enhances radiation resistance, but at concentrations above 5 µM, MnCl_2_ becomes increasingly cytotoxic to the cells (20, 22, 46, 56). *Borrelia burgdorferi* displays a similar Mn-toxicity profile: (i) the near-optimal MnCl_2_ supplementation at the time of inoculation for spirochetes is 0.5 µM in BSK-H medium, but the cells exhibit concentration-dependent toxicity when supplemented with as little as 1 µM MnCl_2_ (Fig. 2); (ii) early-stationary phase cells with a dwindling metabolite pool are highly susceptible to Mn toxicity even with 0.5 µM MnCl_2_ (Fig. 4B, 6D, and 7). Overall, Mn-induced toxicity is absent in log-phase cultures supplemented with 0.5 μM MnCl_2_ (Fig. 3). However, as the spirochetes attain stationary phase and their metabolite pools deplete, Mn toxicity becomes increasingly evident, especially in the ΔMnSOD mutant (Fig. 4). In other organisms, Mn toxicity has been inferred to occur by off-target binding effects of Mn, which may include negatively charged DNA, where it causes helix distortions and decreased genomic integrity, but also by the mismetallation of enzymes and ROS production (Text S2). However, Mn toxification mechanisms generally remain ill-defined across prokaryotes and eukaryotes (28, 29, 41, 46, 99, 102–106).

### Therapeutic opportunities

These findings highlight not only the enhanced recovery conferred by Mn accumulation in *B. burgdorferi* but also the risks associated with Mn overaccumulation, particularly in the ΔMnSOD mutant. As the mutant transitions from exponential to stationary state and its metabolite reserves become depleted, the pool of antioxidant H-Mn shrinks, and then small variations in cellular Mn levels from the optimum will cause shifts between insufficient Mn^2+^, which would impair the antioxidant response, and excessive Mn^2+^, leading to toxicity in metabolite-depleted, starving cells.

While Mn is limited in the host, wild-type *B. burgdorferi* must maintain a delicate balance (safe zone) between too-little and too-much Mn, requiring tight regulation of Mn²^+^ uptake, availability, and buffering by enzymes such as MnSOD, along with adequate accumulation of small metabolites. Together, these factors suggest promising new therapeutic targets for combating Lyme disease (28, 85). For example, lactate dehydrogenase (LDH) inhibitors, which prevent the lactate accumulation discussed above and lead to an accumulation of its precursor pyruvate, are predicted to render stationary phase spirochetes even more susceptible to Mn toxicity once the pool of H-Mn-binding lactate is depleted (Fig. 1, 4, and 6). Indeed, lactate’s bidentate coordination (via -COO⁻ and -OH) makes it a far superior Mn²^+^ ligand than pyruvate, which is monodentate as it lacks an -OH group. Thus, *Borrelia*’s dependence on Mn-lactate homeostasis could be an overlooked vulnerability (83, 84). We note that Mn toxicity is similarly predicted for LDH inhibitor-treated cancer cells expressing the Warburg effect (2, 107).

### Resolving the *Borrelia* Mn puzzle

In diverse organisms across the three domains of life, cellular radiation resistance in general scales with H-Mn content, not MnSOD (2). *Borrelia burgdorferi*, however, is an exception to this rule—the wild-type *Borrelia* H-Mn content can be increased 50 times, but its radiation survival limit is nonetheless unchanged (Fig. 3, 4, and 6). At first, this response to MnCl_2_ supplementation appears puzzling: polyploid *B. burgdorferi* is far less radiation resistant than its H-Mn content upon supplementation would suggest. In contrast, *D. radiodurans* is the opposite exception: polyploid *D. radiodurans* is far more radiation resistant than even its exceptionally high H-Mn content would suggest (2, 65).

In both organisms, the paradoxes are partially resolved by considering their unusual genome architectures. *Borrelia* chromosomes are linear and thus predisposed to DSB damage during irradiation (45), whereas, as reported previously (65, 108, 109), *D. radiodurans’* multiple circular chromosomes are condensed and linked together by Holliday junctions, which further facilitate DSB repair post-irradiation in the presence of H-Mn antioxidants that protect repair machinery (19). The final piece to the puzzle of the low radioresistance of *B. borrelia* is the spirochetes’ limited DNA repair machinery (74).

### Conclusions

We manipulated the cellular H-Mn antioxidant content of wild-type and ΔMnSOD *B. burgdorferi* (ML23)—the uniquely Fe-independent, Mn-accumulating bacterium responsible for Lyme disease—through trace-level MnCl_₂_ supplementation in spirochete cultures. We then tracked their H-Mn levels using EPR and the ligation of the H-Mn pool using ENDOR spectroscopy (Fig. 1). Exposure of *B. burgdorferi* to acute γ-irradiation is an analog model for exposure to the respiratory ROS burst deployed by phagocytic cells of the host’s innate immune response against pathogens. Our results show that MnSOD is indispensable for the radiation survivability of these extreme auxotrophic pathogens, despite their accumulation of H-Mn metabolites following Mn supplementation (Fig. 2 and 5). Nonetheless, the accumulated H-Mn antioxidants are vital for counteracting oxidative stress in *B. burgdorferi*, especially in the absence of MnSOD, as in the ΔMnSOD mutants (Fig. 3 and 6), although their effectiveness is limited by several contributing features of the spirochetes—metabolite composition, linear genome architecture, and limited repair machinery.

The absence of Fe-redox chemistry in *B. burgdorferi* supports the idea that the H-Mn pool of this organism primarily functions as a reservoir of labile Mn²^+^ for metalating its Mn-dependent enzymes, including MnSOD. This contrasts with the H-Mn pool in free-living bacteria like *D. radiodurans*, *L. plantarum*, and *B. subtilis,* where H-Mn antioxidants provide protection against Fe-proteome oxidation under conditions where H_₂_O_₂_ enters the cells from the extracellular environment.

Standard biochemical techniques, which disrupt cells, fall short in addressing these questions due to altered Mn²^+^ speciation. However, paramagnetic resonance spectroscopies of substitutionally labile Mn²^+^ in intact *B. burgdorferi* cells, as applied here (Fig. 5 through 7), offer novel insights into the partitioning of Mn-dependent antioxidant defenses between MnSOD and H-Mn. These findings thus extend the understanding of Mn accumulation beyond free-living bacteria, illustrating how it can both protect and toxify cells (Fig. 8). Our results not only deepen our insight into Mn-dependent antioxidant defenses in *B. burgdorferi* but also suggest a potential new strategy for managing Lyme disease—targeting the *spirochete* by inducing Mn toxicity. The broader application of EPR and ENDOR spectroscopies to other Mn-dependent pathogens holds significant promise for developing countermeasures against *Chlamydia trachomatis*, *Neisseria gonorrhoeae*, *Acinetobacter baumannii*, and *Staphylococcus aureus* (45, 66).

## MATERIALS AND METHODS

### Bacteria

Spirochetes were *Borrelia burgdorferi* (ML23) and *B. burgdorferi* (*sodA*^−^) (ΔMnSOD) (67). Free-living bacteria were *Deinococcus radiodurans* (ATCC BAA-816), *Lactobacillus plantarum* (ATCC 14917), *Bacillus subtilis* spores (PS533), and *Escherichia coli* (MG1655) (5, 65). Cells were grown to the early-stationary phase (∼1 × 10^8^ cells/mL) as follows: WT *D. radiodurans* (ATTC BAA-816) and WT *L. plantarum* (ATCC 14917) were grown at 32°C in liquid TGY medium (1% bactotryptone, 0.5% yeast extract, and 0.1% glucose). WT *E. coli* (K-12, MG1655) was inoculated into liquid LB medium (1% bactotryptone, 0.5% yeast extract, and 1% NaCl) and grown at 37°C. Spores of WT *B. subtilis* (PS533) were prepared as previously described on double strength Schaeffer’s glucose agar plates, which were incubated for ∼48 h at 37°C, and the spores were harvested and highly purified (2, 5, 65).

### Spirochete growth, Mn supplementation, and gamma-irradiation

Wild-type *Borrelia burgdorferi* strain ML23 and its isogenic *sodA*^−^ disruption mutant (ΔMnSOD) were inoculated into standard liquid BSK-H medium (Sigma-Aldrich; No. B8291, 2023) (https://www.sigmaaldrich.com/deepweb/assets/sigmaaldrich/product/documents/259/789/b8291dat.pdf?srsltid=AfmBOopK0yPLfIX-eFuw1aP6ahFAwgxyIhlz1UMPdE2PyCzbbun3dPzm) and initially cultured for 4 and 6 days, respectively, reaching a cell density of ∼1 × 10^8^ cells/mL (early-stationary phase) at 37°C (Fig. 1) (45, 67, 110).

Mn supplementation of BSK-H medium was with MnCl_2_·4H_2_O (0, 0.5, 1, 5, 10, and 20 µM), (e.g., Fig. 2A). Aliquots (200 μL) of the WT and ΔMnSOD cultures were stored frozen in a −80°C freezer until they were irradiated acutely on dry ice (−79°C) to the indicated doses of gamma rays (^60^Co), as described in the legends to Fig. 2B and C. To examine the effect of the Mn^2+^ supplementation on EPR and gamma-irradiation on day-0, WT and ΔMnSOD were grown in BSK-H medium with and without 0.5 µM of MnCl_2_ supplementation and harvested at the early-stationary phase (day-4 for WT and day-6 for ΔMnSOD) (Fig. 1). To evaluate the effect during the early-stationary phase, WT and ΔMnSOD were grown in BSK-H medium to the early-stationary phase, then supplemented or not with 0.5 µM MnCl_2_ and harvested 2 days later (day-6 for WT and day-8 for ΔMnSOD) (Fig. 1). For the irradiation experiments, experimental aliquots (200 µL) were stored at −80°C until they were irradiated acutely on dry ice (−79°C) to the indicated doses of gamma rays (^60^Co), as described in the legends to Fig. 3 and 4. The survival of irradiated and control *B. burgdorferi* cells, MnCl_2_-supplemented or not, was determined by dark-field microscopy of serially diluted liquid cultures, where spirochetes were visually monitored for changes in cell number and also for changes in metabolic activity based on motility, as described previously (45, 110). Each of the survival curves presented in Fig. 2 through 4 represents the outcome of two experimental replicate studies conducted in parallel, each initiated with a population of 10^6^ spirochetes. The experimental design generated two data sets in GraphPad Prism 9: a “shared” model was then framed within the parameters of the other, allowing differences to be assessed as nested models. For example, in Fig. 3 and 4, the differences between curves (MnCl_2_-supplemented or not) are displayed relative to this shared framework, showing precisely where statistical distinctions reside. Data were analyzed using an extra sum-of-squares *F*-test (nonlinear regression) in GraphPad Prism 9. A *P*-value ≤0.05 was considered statistically significant. As noted in Results, *B. burgdorferi* cannot be enumerated on plates. Radiation survivability was therefore gauged in liquid culture—a method that necessarily limited the number of replicates we could process over 33 days of continuous monitoring. The sample processing for paramagnetic spectroscopy analysis is described next.

### EPR and ENDOR/ESEEM spectroscopy

For EPR/ENDOR/ESEEM spectroscopy (Fig. 5 through 7), 30 mL early-stationary phase WT and ΔMnSOD *B. burgdorferi* cultures (2–5 × 10^8^ cells/mL) were harvested by centrifugation, and cells were washed twice and resuspended in 0.3 mL of 20% glycerol (vol/vol) nanopure water (MilliQ H_2_O from “Banstead Nano Pure Diamond” [Thermo Scientific]). EPR tubes (quartz, inner diameter 2.1 mm, length 65–70 mm, Wilmad-LabGlass) were filled with ∼80 µL of the concentrated cell suspensions, then frozen on dry ice, sterilized (10 kGy), and stored at −80°C for EPR analyses (45, 48).

Absorption display 35 GHz continuous-wave EPR spectra were recorded using a lab-built EPR spectrometer described in detail (2, 5, 45, 65). Experimental conditions: MW frequency, 34.9 GHz; temperature, 2 K; modulation amplitude, 2 G; time constant, 64 ms; and scan rate, 1 kG/min. Data acquisition was done by using a home-written program in LabVIEW. EPR spectra were simulated with EasySpin software (6.0.6) (111). The Mn^2+^ EPR spectrum could be partitioned into the percentage of a narrow component, associated with antioxidant metabolite complexes of Mn^2+^ (ligands of phosphates, imidazole, and carboxylates), designated H-Mn, and that of a broad component associated with Mn-enzymes, including MnSOD2, denoted L-Mn. The H-Mn EPR displays six sharp peaks at the center (due to ^55^Mn electron-nuclear hyperfine splittings) with narrow wings that are reproduced with a small zero-field splitting parameter, *D* ~ 1,000 MHz/0.03 cm^−1^. The broad L-Mn component is simulated with *D* ~ 4,000 MHz/0.12 cm^−1^. For further details of the procedures for determining the fractional contributions of H-Mn and L-Mn to the Mn^2+^ EPR spectra, see references 2, 5, 45, 65.

Pulsed EPR and ENDOR/ESEEM experiments were performed on a lab-built Q-band 35 GHz pulse spectrometer (112) that employs SpecMan4EPR (113) for data collection. Pulsed ESE-EPR employed a two-pulse echo sequence -π/2-*τ*-π-*τ*-echo. Experimental conditions were as follows: (π/2) = 60 ns; *t* = 500 ns; repetition time = 10 ms; scan range, 1–16 kG; and scan rate, 1 kG/min. ^1^H Davies ENDOR spectra employ three-pulse sequence π-Trf-π/2-*τ*-π-*τ*-echo, π/2 = 60 ns, *τ* = 500 ns, Trf = 20 µs, and magnetic field *~* 12.5 kG. ^31^P ENDOR spectra were collected using refocused Mims four-pulse sequence (114) with typical experimental conditions, magnetic field *~* 12.5 kG ; MW frequency, 34.769 GHz; ReMims pulse sequence, π/2-*τ*1-π/2-T-π/2-τ2--π-(τ1+ τ2)-echo, where (π/2) = 30 ns; *t* = 120 ns; RF pulse, *T* = 20 μs; τ1 = 200 ns; τ2 = 400 ns; repetition time = 10 ms; *T* = 2 K. The ENDOR spectra are normalized to their Mn^2+^ EPR intensity and thus ^31^P ENDOR peaks’ amplitude is a direct measure of bound Pi (Fig. 7). Three-Pulse ESEEM spectra were recorded using the pulse sequence, π/2-τ-π/2-T-π/2-τ – echo, where *T* is the time varied between second and third microwave pulses, with four-step phase cycling to suppress unwanted Hahn and refocused echoes (115). A ^14^N nucleus (*I* = 1) directly coordinated with ^55^Mn creates modulation in the electron spin echo decay generated by ^14^N hyperfine and quadrupolar interactions (115). To quantitate ^14^N ESEEM responses from cellular Mn^2+^, we chose the ^14^N response from the Mn-imidazole complex as a standard, which binds one imidazole and (presumably) five waters. ESEEM conditions: magnetic field ~ 12.5 kG*; t_π/2_ =* 50 ns; *t* = 400 ns; and repetition time, 10 ms.

### Highlights

Outcomes of this investigation include the following:

- EPR and ENDOR spectroscopies reveal Mn^2+^ partitioning between enzyme-bound (L-Mn) and metabolite-bound (H-Mn) pools and monitor H-Mn speciation.
- MnSOD defends *B. burgdorferi* against oxidative assault by extracellular O_2_^•−^, and H-Mn provides cytoplasmic protection.
- MnCl_₂_ supplementation of metabolite-replete cells complements ΔMnSOD.
- Mn toxification is a potential therapeutic strategy against Lyme disease.

## SUPPLEMENTAL MATERIAL

Fig. S1 mbio.02824-25-s0001.eps Partitioning of Mn^2+^ EPR spectra of WT and *sodA^−^* day 0 *Borrelia* cells.

Fig. S2 mbio.02824-25-s0002.eps Mn supplementation: partitioning of Mn^2+^ EPR spectra of WT day 0 *Borrelia* cells.

Fig. S3 mbio.02824-25-s0003.eps Mn supplementation: partitioning of Mn^2+^ EPR spectra of ΔMnSOD day 0 *Borrelia* cells.

Fig. S4 mbio.02824-25-s0004.eps Mn supplementation: partitioning of Mn^2+^ EPR spectra of WT day 4 *Borrelia* cells.

Fig. S5 mbio.02824-25-s0005.eps Mn supplementation: partitioning of Mn^2+^ EPR spectra of ΔMnSOD day 6 *Borrelia* cells.

Fig. S6 mbio.02824-25-s0006.eps *Borrelia* antioxidants H-Mn have no nitrogenous ligands bound.

Supplemental material mbio.02824-25-s0007.docx Texts S1 and S2 and legends for Fig. S1-S6.

ASM does not own the copyrights to Supplemental Material that may be linked to, or accessed through, an article. The authors have granted ASM a non-exclusive, world-wide license to publish the Supplemental Material files. Please contact the corresponding author directly for reuse.

## References

[1] McNaughton RL, Reddi AR, Clement MHS, Sharma A, Barnese K, Rosenfeld L, Gralla EB, Valentine JS, Culotta VC, Hoffman BM. 2010. Probing *in vivo* Mn^2+^ speciation and oxidative stress resistance in yeast cells with electron-nuclear double resonance spectroscopy. Proc Natl Acad Sci USA 107:15335–15339. doi:10.1073/pnas.1009648107 20702768 PMC2932569

[2] Sharma A, Gaidamakova EK, Grichenko O, Matrosova VY, Hoeke V, Klimenkova P, Conze IH, Volpe RP, Tkavc R, Gostinčar C, Gunde-Cimerman N, DiRuggiero J, Shuryak I, Ozarowski A, Hoffman BM, Daly MJ. 2017. Across the tree of life, radiation resistance is governed by antioxidant Mn^2+^, gauged by paramagnetic resonance. Proc Natl Acad Sci USA 114:E9253–E9260. doi:10.1073/pnas.1713608114 29042516 PMC5676931

[3] Slade D, Radman M. 2011. Oxidative stress resistance in *Deinococcus radiodurans*. Microbiol Mol Biol Rev 75:133–191. doi:10.1128/MMBR.00015-10 21372322 PMC3063356

[4] Culotta VC, Daly MJ. 2013. Manganese complexes: diverse metabolic routes to oxidative stress resistance in prokaryotes and yeast. Antioxid Redox Signal 19:933–944. doi:10.1089/ars.2012.5093 23249283 PMC3763226

[5] Gaidamakova EK, Sharma A, Matrosova VY, Grichenko O, Volpe RP, Tkavc R, Conze IH, Klimenkova P, Balygina I, Horne WH, Gostinčar C, Chen X, Makarova KS, Shuryak I, Srinivasan C, Jackson-Thompson B, Hoffman BM, Daly MJ. 2022. Small-molecule Mn antioxidants in *Caenorhabditis elegans* and *Deinococcus radiodurans* supplant MnSOD enzymes during aging and irradiation. mBio 13:e03394-21. doi:10.1128/mbio.03394-21 35012337 PMC8749422

[6] Anjem A, Varghese S, Imlay JA. 2009. Manganese import is a key element of the OxyR response to hydrogen peroxide in *Escherichia coli*. Mol Microbiol 72:844–858. doi:10.1111/j.1365-2958.2009.06699.x 19400769 PMC2776087

[7] Anjem A, Imlay JA. 2012. Mononuclear iron enzymes are primary targets of hydrogen peroxide stress. J Biol Chem 287:15544–15556. doi:10.1074/jbc.M111.330365 22411989 PMC3346116

[8] Berlett BS, Levine RL. 2014. Designing antioxidant peptides. Redox Rep 19:80–86. doi:10.1179/1351000213Y.0000000078 24520968 PMC4130572

[9] Čapek J, Večerek B. 2023. Why is manganese so valuable to bacterial pathogens? Front Cell Infect Microbiol 13:943390. doi:10.3389/fcimb.2023.943390 36816586 PMC9936198

[10] Rohaun SK, Sethu R, Imlay JA. 2024. Microbes vary strategically in their metalation of mononuclear enzymes. Proc Natl Acad Sci USA 121:e2401738121. doi:10.1073/pnas.2401738121 38743623 PMC11127058

[11] Archibald FS, Fridovich I. 1981. Manganese and defenses against oxygen toxicity in *Lactobacillus plantarum*. J Bacteriol 145:442–451. doi:10.1128/jb.145.1.442-451.1981 6257639 PMC217292

[12] Archibald FS, Fridovich I. 1981. Manganese, superoxide dismutase, and oxygen tolerance in some lactic acid bacteria. J Bacteriol 146:928–936. doi:10.1128/jb.146.3.928-936.1981 6263860 PMC216946

[13] Stadtman ER, Berlett BS, Chock PB. 1990. Manganese-dependent disproportionation of hydrogen peroxide in bicarbonate buffer. Proc Natl Acad Sci USA 87:384–388. doi:10.1073/pnas.87.1.384 2296593 PMC53268

[14] Barnese K, Gralla EB, Valentine JS, Cabelli DE. 2012. Biologically relevant mechanism for catalytic superoxide removal by simple manganese compounds. Proc Natl Acad Sci USA 109:6892–6897. doi:10.1073/pnas.1203051109 22505740 PMC3344976

[15] Daly MJ, Gaidamakova EK, Matrosova VY, Kiang JG, Fukumoto R, Lee DY, Wehr NB, Viteri GA, Berlett BS, Levine RL. 2010. Small-molecule antioxidant proteome-shields in *Deinococcus radiodurans*. PLoS One 5:e12570. doi:10.1371/journal.pone.0012570 20838443 PMC2933237

[16] Krisko A, Radman M. 2010. Protein damage and death by radiation in *Escherichia coli* and *Deinococcus radiodurans*. Proc Natl Acad Sci USA 107:14373–14377. doi:10.1073/pnas.1009312107 20660760 PMC2922536

[17] Daly MJ. 2012. Death by protein damage in irradiated cells. DNA Repair (Amsterdam) 11:12–21. doi:10.1016/j.dnarep.2011.10.024 22112864

[18] Yang H, Sharma A, Daly MJ, Hoffman BM. 2024. The ternary complex of Mn^2+^, synthetic decapeptide DP1 (DEHGTAVMLK), and orthophosphate is a superb antioxidant. Proc Natl Acad Sci USA 121:e2417389121. doi:10.1073/pnas.2417389121 39665753 PMC11665895

[19] Daly MJ. 2023. The scientific revolution that unraveled the astonishing DNA repair capacity of the *Deinococcaceae*: 40 years on. Can J Microbiol 69:369–386. doi:10.1139/cjm-2023-0059 37267626

[20] Daly MJ, Gaidamakova EK, Matrosova VY, Vasilenko A, Zhai M, Venkateswaran A, Hess M, Omelchenko MV, Kostandarithes HM, Makarova KS, Wackett LP, Fredrickson JK, Ghosal D. 2004. Accumulation of Mn(II) in *Deinococcus radiodurans* facilitates gamma-radiation resistance. Science 306:1025–1028. doi:10.1126/science.1103185 15459345

[21] Fredrickson JK, Li SW, Gaidamakova EK, Matrosova VY, Zhai M, Sulloway HM, Scholten JC, Brown MG, Balkwill DL, Daly MJ. 2008. Protein oxidation: key to bacterial desiccation resistance? ISME J 2:393–403. doi:10.1038/ismej.2007.116 18273068

[22] Daly MJ, Gaidamakova EK, Matrosova VY, Vasilenko A, Zhai M, Leapman RD, Lai B, Ravel B, Li SMW, Kemner KM, Fredrickson JK. 2007. Protein oxidation implicated as the primary determinant of bacterial radioresistance. PLoS Biol 5:e92. doi:10.1371/journal.pbio.0050092 17373858 PMC1828145

[23] Daly MJ. 2009. A new perspective on radiation resistance based on *Deinococcus radiodurans*. Nat Rev Microbiol 7:237–245. doi:10.1038/nrmicro2073 19172147

[24] Setlow P, Christie G. 2023. New thoughts on an old topic: secrets of bacterial spore resistance slowly being revealed. Microbiol Mol Biol Rev 87:e00080-22. doi:10.1128/mmbr.00080-22 36927044 PMC10304885

[25] Helmann JD. 2025. Metals in motion: understanding labile metal pools in bacteria. Biochemistry 64:329–345. doi:10.1021/acs.biochem.4c00726 39755956 PMC11755726

[26] Kehres DG, Maguire ME. 2003. Emerging themes in manganese transport, biochemistry and pathogenesis in bacteria. FEMS Microbiol Rev 27:263–290. doi:10.1016/S0168-6445(03)00052-4 12829271

[27] Aguirre JD, Clark HM, McIlvin M, Vazquez C, Palmere SL, Grab DJ, Seshu J, Hart PJ, Saito M, Culotta VC. 2013. A manganese-rich environment supports superoxide dismutase activity in a Lyme disease pathogen, *Borrelia burgdorferi*. J Biol Chem 288:8468–8478. doi:10.1074/jbc.M112.433540 23376276 PMC3605662

[28] Martin JE, Waters LS. 2022. Regulation of bacterial manganese homeostasis and usage during stress responses and pathogenesis. Front Mol Biosci 9:945724. doi:10.3389/fmolb.2022.945724 35911964 PMC9334652

[29] Wang X, Zhang F, Dai Q, Zhao Y, Liu M, Wu C, Tang J, Gu Y, Xie Z, Chen S, Zhang M, Luo C, Wang X, Wang Y, Shen X, Xu L. 2025. Manganese transport systems of two enteric bacteria enhance their resistance to stress and enable the bacteria to evade the innate immune responses. Cytokine 194:157009. doi:10.1016/j.cyto.2025.157009 40795753

[30] Burgdorfer W, Barbour AG, Hayes SF, Benach JL, Grunwaldt E, Davis JP. 1982. Lyme disease-a tick-borne spirochetosis? Science 216:1317–1319. doi:10.1126/science.7043737 7043737

[31] Bourret TJ, Boyle WK, Zalud AK, Valenzuela JG, Oliveira F, Lopez JE. 2019. The relapsing fever spirochete *Borrelia turicatae* persists in the highly oxidative environment of its soft-bodied tick vector. Cell Microbiol 21:e12987. doi:10.1111/cmi.12987 30489694 PMC6454574

[32] Uppalapati SR, Vazquez-Torres A. 2022. Manganese utilization in *Salmonella* pathogenesis: beyond the canonical antioxidant response. Front Cell Dev Biol 10:924925. doi:10.3389/fcell.2022.924925 35903545 PMC9315381

[33] Bourgeois JS, Hu LT. 2024. Hitchhiker’s guide to *Borrelia burgdorferi*. J Bacteriol 206:e00116-24. doi:10.1128/jb.00116-24 39140751 PMC11411949

[34] Corona A, Schwartz I. 2015. Borrelia burgdorferi: Carbon metabolism and the tick-mammal enzootic cycle. Microbiol Spectr 3:1–14. doi:10.1128/microbiolspec.MBP-0011-2014 PMC7942402 26185064

[35] Broxton CN, Culotta VC. 2016. SOD enzymes and microbial pathogens: surviving the oxidative storm of infection. PLoS Pathog 12:e1005295. doi:10.1371/journal.ppat.1005295 26742105 PMC4712152

[36] Murdoch CC, Skaar EP. 2022. Nutritional immunity: the battle for nutrient metals at the host-pathogen interface. Nat Rev Microbiol 20:657–670. doi:10.1038/s41579-022-00745-6 35641670 PMC9153222

[37] Andrés CMC, Pérez de la Lastra JM, Andrés Juan C, Plou FJ, Pérez-Lebeña E. 2023. Superoxide anion chemistry—its role at the core of the innate immunity. Int J Mol Sci 24:1841. doi:10.3390/ijms24031841 36768162 PMC9916283

[38] Imlay JA. 2025. The barrier properties of biological membranes dictate how cells experience oxidative stress. Mol Microbiol 123:454–463. doi:10.1111/mmi.15353 40091849 PMC12051229

[39] Lynch RE, Fridovich I. 1978. Permeation of the erythrocyte stroma by superoxide radical. J Biol Chem 253:4697–4699. doi:10.1016/S0021-9258(17)30446-5 207707

[40] Liochev SI, Fridovich I. 2005. Cross-compartment protection by SOD1. Free Radic Biol Med 38:146–147. doi:10.1016/j.freeradbiomed.2004.10.017 15589383

[41] Ghosal D, Omelchenko MV, Gaidamakova EK, Matrosova VY, Vasilenko A, Venkateswaran A, Zhai M, Kostandarithes HM, Brim H, Makarova KS, Wackett LP, Fredrickson JK, Daly MJ. 2005. How radiation kills cells: survival of *Deinococcus radiodurans* and *Shewanella oneidensis* under oxidative stress. FEMS Microbiol Rev 29:361–375. doi:10.1016/j.femsre.2004.12.007 15808748

[42] Shuryak I, Matrosova VY, Gaidamakova EK, Tkavc R, Grichenko O, Klimenkova P, Volpe RP, Daly MJ. 2017. Microbial cells can cooperate to resist high-level chronic ionizing radiation. PLoS One 12:e0189261. doi:10.1371/journal.pone.0189261 29261697 PMC5738026

[43] Esteve-Gassent MD, Smith TC, Small CM, Thomas DP, Seshu J. 2015. Absence of *sodA* increases the levels of oxidation of key metabolic determinants of *Borrelia burgdorferi*. PLoS One 10:e0136707. doi:10.1371/journal.pone.0136707 26322513 PMC4556403

[44] Zückert WR. 2019. Protein secretion in spirochetes. Microbiol Spectr 7:1–10. doi:10.1128/microbiolspec.psib-0026-2019 PMC6579041 31198130

[45] Londoño AF, Sharma A, Sealy J, Rana VS, Foor SD, Matrosova VY, Gaidamakova EK, Volpe RP, Daly MJ, Hoffman BM, Pal U, Dumler JS. 2025. *Borrelia burgdorferi* radiosensitivity and Mn antioxidant content: antigenic preservation and pathobiology. mBio 16:e03131-24. doi:10.1128/mbio.03131-24 39727419 PMC11796347

[46] Chou FI, Tan ST. 1990. Manganese(II) induces cell division and increases in superoxide dismutase and catalase activities in an aging deinococcal culture. J Bacteriol 172:2029–2035. doi:10.1128/jb.172.4.2029-2035.1990 2318808 PMC208701

[47] Markillie LM, Varnum SM, Hradecky P, Wong KK. 1999. Targeted mutagenesis by duplication insertion in the radioresistant bacterium *Deinococcus radiodurans*: radiation sensitivities of catalase (*katA*) and superoxide dismutase (*sodA*) mutants. J Bacteriol 181:666–669. doi:10.1128/JB.181.2.666-669.1999 9882685 PMC93425

[48] Sharma A, Gaidamakova EK, Matrosova VY, Bennett B, Daly MJ, Hoffman BM. 2013. Responses of Mn^2+^ speciation in *Deinococcus radiodurans* and *Escherichia coli* to γ-radiation by advanced paramagnetic resonance methods. Proc Natl Acad Sci USA 110:5945–5950. doi:10.1073/pnas.1303376110 23536297 PMC3625348

[49] Santos SP, Mitchell EP, Franquelim HG, Castanho MARB, Abreu IA, Romão CV. 2015. Dps from *Deinococcus radiodurans*: oligomeric forms of Dps1 with distinct cellular functions and Dps2 involved in metal storage. FEBS J 282:4307–4327. doi:10.1111/febs.13420 26290287

[50] Santos SP, Yang Y, Rosa MTG, Rodrigues MAA, De La Tour CB, Sommer S, Teixeira M, Carrondo MA, Cloetens P, Abreu IA, Romão CV. 2019. The interplay between Mn and Fe in *Deinococcus radiodurans* triggers cellular protection during paraquat-induced oxidative stress. Sci Rep 9:17217. doi:10.1038/s41598-019-53140-2 31748604 PMC6868200

[51] Peana M, Gumienna-Kontecka E, Piras F, Ostrowska M, Piasta K, Krzywoszynska K, Medici S, Zoroddu MA. 2020. Exploring the specificity of rationally designed peptides reconstituted from the cell-free extract of *Deinococcus radiodurans* toward Mn(II) and Cu(II). Inorg Chem 59:4661–4684. doi:10.1021/acs.inorgchem.9b03737 32212645 PMC7467671

[52] Scott MD, Meshnick SR, Eaton JW. 1989. Superoxide dismutase amplifies organismal sensitivity to ionizing radiation. J Biol Chem 264:2498–2501. doi:10.1016/S0021-9258(19)81641-1 2644263

[53] Sukhi SS, Shashidhar R, Kumar SA, Bandekar JR. 2009. Radiation resistance of *Deinococcus radiodurans* R1 with respect to growth phase. FEMS Microbiol Lett 297:49–53. doi:10.1111/j.1574-6968.2009.01652.x 19490129

[54] Caffrey BJ, Pedrazo-Tardajos A, Liberti E, Gaunt B, Kim JS, Kirkland AI. 2024. Liquid phase electron microscopy of bacterial ultrastructure. Small 20:e2402871. doi:10.1002/smll.202402871 39239997 PMC11636060

[55] Rao NN, Liu S, Kornberg A. 1998. Inorganic polyphosphate in *Escherichia coli*: the phosphate regulon and the stringent response. J Bacteriol 180:2186–2193. doi:10.1128/JB.180.8.2186-2193.1998 9555903 PMC107147

[56] Venkateswaran A, McFarlan SC, Ghosal D, Minton KW, Vasilenko A, Makarova K, Wackett LP, Daly MJ. 2000. Physiologic determinants of radiation resistance in *Deinococcus radiodurans*. Appl Environ Microbiol 66:2620–2626. doi:10.1128/AEM.66.6.2620-2626.2000 10831446 PMC110589

[57] Bugrysheva J, Dobrikova EY, Sartakova ML, Caimano MJ, Daniels TJ, Radolf JD, Godfrey HP, Cabello FC. 2003. Characterization of the stringent response and rel(Bbu) expression in *Borrelia burgdorferi*. J Bacteriol 185:957–965. doi:10.1128/JB.185.3.957-965.2003 12533471 PMC142832

[58] Li X, Pal U, Ramamoorthi N, Liu X, Desrosiers DC, Eggers CH, Anderson JF, Radolf JD, Fikrig E. 2007. The Lyme disease agent *Borrelia burgdorferi* requires BB0690, a Dps homologue, to persist within ticks. Mol Microbiol 63:694–710. doi:10.1111/j.1365-2958.2006.05550.x 17181780

[59] Posey JE, Gherardini FC. 2000. Lack of a role for iron in the Lyme disease pathogen. Science 288:1651–1653. doi:10.1126/science.288.5471.1651 10834845

[60] Troxell B, Ye M, Yang Y, Carrasco SE, Lou Y, Yang XF. 2013. Manganese and zinc regulate virulence determinants in *Borrelia burgdorferi*. Infect Immun 81:2743–2752. doi:10.1128/IAI.00507-13 23690398 PMC3719580

[61] Tsednee M, Castruita M, Salomé PA, Sharma A, Lewis BE, Schmollinger SR, Strenkert D, Holbrook K, Otegui MS, Khatua K, Das S, Datta A, Chen S, Ramon C, Ralle M, Weber PK, Stemmler TL, Pett-Ridge J, Hoffman BM, Merchant SS. 2019. Manganese co-localizes with calcium and phosphorus in *Chlamydomonas acidocalcisomes* and is mobilized in manganese-deficient conditions. J Biol Chem 294:17626–17641. doi:10.1074/jbc.RA119.009130 31527081 PMC6873200

[62] Lingappa UF, Yeager CM, Sharma A, Lanza NL, Morales DP, Xie G, Atencio AD, Chadwick GL, Monteverde DR, Magyar JS, Webb SM, Valentine JS, Hoffman BM, Fischer WW. 2021. An ecophysiological explanation for manganese enrichment in rock varnish. Proc Natl Acad Sci USA 118:e2025188118. doi:10.1073/pnas.2025188118 34161271 PMC8237629

[63] Seeler JF, Sharma A, Zaluzec NJ, Bleher R, Lai B, Schultz EG, Hoffman BM, LaBonne C, Woodruff TK, O’Halloran TV. 2021. Metal ion fluxes controlling amphibian fertilization. Nat Chem 13:683–691. doi:10.1038/s41557-021-00705-2 34155376 PMC8475775

[64] Volpe RP, Sen A, Sharma A, Kathiresan V, Hoffman BM, Cox RT. 2025. Prophylactically feeding manganese to *Drosophila* confers sex-specific protection from acute ionizing radiation independent of MnSOD2 levels. Antioxidants (Basel) 14:134. doi:10.3390/antiox14020134 40002321 PMC11851552

[65] Horne WH, Volpe RP, Korza G, DePratti S, Conze IH, Shuryak I, Grebenc T, Matrosova VY, Gaidamakova EK, Tkavc R, Sharma A, Gostinčar C, Gunde-Cimerman N, Hoffman BM, Setlow P, Daly MJ. 2022. Effects of desiccation and freezing on microbial ionizing radiation survivability: considerations for Mars sample return. Astrobiology 22:1337–1350. doi:10.1089/ast.2022.0065 36282180 PMC9618380

[66] Broder KC, Matrosova VY, Tkavc R, Gaidamakova EK, Ho L, Macintyre AN, Soc A, Diallo A, Darnell SC, Bash S, Daly MJ, Jerse AE, Liechti GW. 2024. Irradiated whole cell *Chlamydia* vaccine confers significant protection in a murine genital tract challenge model. NPJ Vaccines 9:207. doi:10.1038/s41541-024-00968-z 39528548 PMC11554809

[67] Esteve-Gassent MD, Elliott NL, Seshu J. 2009. *sodA* is essential for virulence of *Borrelia burgdorferi* in the murine model of Lyme disease. Mol Microbiol 71:594–612. doi:10.1111/j.1365-2958.2008.06549.x 19040638

[68] Azzam EI, Jay-Gerin JP, Pain D. 2012. Ionizing radiation-induced metabolic oxidative stress and prolonged cell injury. Cancer Lett 327:48–60. doi:10.1016/j.canlet.2011.12.012 22182453 PMC3980444

[69] Zhang J, Takacs CN, McCausland JW, Mueller EA, Buron J, Thappeta Y, Wachter J, Rosa PA, Jacobs-Wagner C. 2025. *Borrelia burgdorferi* loses essential genetic elements and cell proliferative potential during stationary phase in culture but not in the tick vector. J Bacteriol 207:e00457-24. doi:10.1128/jb.00457-24 39950812 PMC11925233

[70] Dutta S, Rana VS, Backstedt BT, Shakya AK, Kitsou C, Yas OB, Smith AA, Ronzetti MH, Lipman RM, Araujo-Aris S, Yang X, Rai G, Lin YP, Herzberg O, Pal U. 2025. Borrelial phosphomannose isomerase as a cell surface localized protein that retains enzymatic activity and promotes host-pathogen interaction. mBio 16:e03609-24. doi:10.1128/mbio.03609-24 39932273 PMC11898738

[71] Archibald FS, Duong MN. 1984. Manganese acquisition by *Lactobacillus plantarum*. J Bacteriol 158:1–8. doi:10.1128/jb.158.1.1-8.1984 6715278 PMC215370

[72] Ghosh S, Ramirez-Peralta A, Gaidamakova E, Zhang P, Li YQ, Daly MJ, Setlow P. 2011. Effects of Mn levels on resistance of *Bacillus megaterium* spores to heat, radiation and hydrogen peroxide. J Appl Microbiol 111:663–670. doi:10.1111/j.1365-2672.2011.05095.x 21714839

[73] Granger AC, Gaidamakova EK, Matrosova VY, Daly MJ, Setlow P. 2011. Effects of Mn and Fe levels on *Bacillus subtilis* spore resistance and effects of Mn^2+^, other divalent cations, orthophosphate, and dipicolinic acid on protein resistance to ionizing radiation. Appl Environ Microbiol 77:32–40. doi:10.1128/AEM.01965-10 21057011 PMC3019732

[74] Hardy PO, Chaconas G. 2013. The nucleotide excision repair system of *Borrelia burgdorferi* is the sole pathway involved in repair of DNA damage by UV light. J Bacteriol 195:2220–2231. doi:10.1128/JB.00043-13 23475971 PMC3650546

[75] Huang SH, Hart MA, Wade M, Cozart MR, McGrath SL, Kobryn K. 2017. Biochemical characterization of *Borrelia burgdorferi*’s RecA protein. PLoS One 12:e0187382. doi:10.1371/journal.pone.0187382 29088268 PMC5663514

[76] Holly KJ, Kataria A, Flaherty DP, Groshong AM. 2024. Unguarded liabilities: *Borrelia burgdorferi’s* complex amino acid dependence exposes unique avenues of inhibition. Front Antibiot 3:1395425. doi:10.3389/frabi.2024.1395425 39816271 PMC11732028

[77] Liu Y, Zhou J, Omelchenko MV, Beliaev AS, Venkateswaran A, Stair J, Wu L, Thompson DK, Xu D, Rogozin IB, Gaidamakova EK, Zhai M, Makarova KS, Koonin EV, Daly MJ. 2003. Transcriptome dynamics of *Deinococcus radiodurans* recovering from ionizing radiation. Proc Natl Acad Sci USA 100:4191–4196. doi:10.1073/pnas.0630387100 12651953 PMC153069

[78] Keele BB, McCord JM, Fridovich I. 1971. Further characterization of bovine superoxide dismutase and its isolation from bovine heart. J Biol Chem 246:2875–2880. doi:10.1016/S0021-9258(18)62263-X 4324341

[79] Imlay JA. 2011. Redox pioneer: professor Irwin Fridovich. Antioxid Redox Signal 14:335–340. doi:10.1089/ars.2010.3264 20518701 PMC3026652

[80] Tobin GJ, Tobin JK, Gaidamakova EK, Wiggins TJ, Bushnell RV, Lee W-M, Matrosova VY, Dollery SJ, Meeks HN, Kouiavskaia D, Chumakov K, Daly MJ. 2020. A novel gamma radiation-inactivated sabin-based polio vaccine. PLoS One 15:e0228006. doi:10.1371/journal.pone.0228006 31999745 PMC6991977

[81] Inaoka T, Matsumura Y, Tsuchido T. 1999. SodA and manganese are essential for resistance to oxidative stress in growing and sporulating cells of *Bacillus subtilis*. J Bacteriol 181:1939–1943. doi:10.1128/JB.181.6.1939-1943.1999 10074093 PMC93599

[82] Bosma EF, Rau MH, van Gijtenbeek LA, Siedler S. 2021. Regulation and distinct physiological roles of manganese in bacteria. FEMS Microbiol Rev 45:fuab028. doi:10.1093/femsre/fuab028 34037759 PMC8632737

[83] Lynch A, Pearson P, Savinov SN, Li AY, Rich SM. 2023. Lactate dehydrogenase inhibitors suppress *Borrelia burgdorferi* growth *in vitro*. Pathogens 12:962. doi:10.3390/pathogens12070962 37513809 PMC10384987

[84] Sze CW, Lynch MJ, Zhang K, Neau DB, Ealick SE, Crane BR, Li C. 2025. Lactate dehydrogenase is the Achilles’ heel of Lyme disease bacterium *Borreliella burgdorferi*. mBio 16:e03728-24. doi:10.1128/mbio.03728-24 40111021 PMC11980376

[85] Waters LS. 2020. Bacterial manganese sensing and homeostasis. Curr Opin Chem Biol 55:96–102. doi:10.1016/j.cbpa.2020.01.003 32086169 PMC9997548

[86] Mattimore V, Battista JR. 1996. Radioresistance of *Deinococcus radiodurans*: functions necessary to survive ionizing radiation are also necessary to survive prolonged desiccation. J Bacteriol 178:633–637. doi:10.1128/jb.178.3.633-637.1996 8550493 PMC177705

[87] Bruce AK, Berner JD. 1976. Respiratory activity as a determinant of radiation survival response. Can J Microbiol 22:1336–1344. doi:10.1139/m76-197 788873

[88] Kerstholt M, Netea MG, Joosten LAB. 2020. *Borrelia burgdorferi* hijacks cellular metabolism of immune cells: consequences for host defense. Ticks Tick Borne Dis 11:101386. doi:10.1016/j.ttbdis.2020.101386 32035898

[89] Imlay JA. 2006. Iron-sulphur clusters and the problem with oxygen. Mol Microbiol 59:1073–1082. doi:10.1111/j.1365-2958.2006.05028.x 16430685

[90] Brim H, Osborne JP, Kostandarithes HM, Fredrickson JK, Wackett LP, Daly MJ. 2006. *Deinococcus radiodurans* engineered for complete toluene degradation facilitates Cr(VI) reduction. Microbiology (Reading) 152:2469–2477. doi:10.1099/mic.0.29009-0 16849809

[91] Fraser CM, Casjens S, Huang WM, Sutton GG, Clayton R, Lathigra R, White O, Ketchum KA, Dodson R, Hickey EK, et al.. 1997. Genomic sequence of a Lyme disease spirochaete, *Borrelia burgdorferi*. Nature 390:580–586. doi:10.1038/37551 9403685

[92] Makarova KS, Aravind L, Wolf YI, Tatusov RL, Minton KW, Koonin EV, Daly MJ. 2001. Genome of the extremely radiation-resistant bacterium *Deinococcus radiodurans* viewed from the perspective of comparative genomics. Microbiol Mol Biol Rev 65:44–79. doi:10.1128/MMBR.65.1.44-79.2001 11238985 PMC99018

[93] Han R, Fang J, Jiang J, Gaidamakova EK, Tkavc R, Daly MJ, Contreras LM. 2020. Signal recognition particle RNA contributes to oxidative stress response in *Deinococcus radiodurans* by modulating catalase localization. Front Microbiol 11:613571. doi:10.3389/fmicb.2020.613571 33391243 PMC7775534

[94] Archibald F. 1986. Manganese: its acquisition by and function in the lactic acid bacteria. Crit Rev Microbiol 13:63–109. doi:10.3109/10408418609108735 3522109

[95] Sanchez RJ, Srinivasan C, Munroe WH, Wallace MA, Martins J, Kao TY, Le K, Gralla EB, Valentine JS. 2005. Exogenous manganous ion at millimolar levels rescues all known dioxygen-sensitive phenotypes of yeast lacking CuZnSOD. J Biol Inorg Chem 10:913–923. doi:10.1007/s00775-005-0044-y 16283393

[96] Lin YT, Hoang H, Hsieh SI, Rangel N, Foster AL, Sampayo JN, Lithgow GJ, Srinivasan C. 2006. Manganous ion supplementation accelerates wild type development, enhances stress resistance, and rescues the life span of a short–lived *Caenorhabditis elegans* mutant. Free Radic Biol Med 40:1185–1193. doi:10.1016/j.freeradbiomed.2005.11.007 16545686

[97] Hood MN, Ayompe E, Holmes-Hampton GP, Korotcov A, Wuddie K, Aschenake Z, Ahmed AE, Creavalle M, Knollmann-Ritschel B. 2024. Preliminary promising findings for manganese chloride as a novel radiation countermeasure against acute radiation syndrome. Mil Med 189:598–607. doi:10.1093/milmed/usae198 39160887

[98] Webb KM, Yu J, Robinson CK, Noboru T, Lee YC, DiRuggiero J. 2013. Effects of intracellular Mn on the radiation resistance of the halophilic archaeon *Halobacterium salinarum*. Extremophiles 17:485–497. doi:10.1007/s00792-013-0533-9 23532412

[99] Gupta P, Gayen M, Smith JT, Gaidamakova EK, Matrosova VY, Grichenko O, Knollmann-Ritschel B, Daly MJ, Kiang JG, Maheshwari RK. 2016. MDP: a *Deinococcus* Mn^2+^-decapeptide complex protects mice from ionizing radiation. PLoS One 11:e0160575. doi:10.1371/journal.pone.0160575 27500529 PMC4976947

[100] Chen H, Li Y, Li H, Chen X, Fu H, Mao D, Chen W, Lan L, Wang C, Hu K, Li J, Zhu C, Evans I, Cheung E, Lu D, He Y, Behrens A, Yin D, Zhang C. 2024. NBS1 lactylation is required for efficient DNA repair and chemotherapy resistance. Nature 631:663–669. doi:10.1038/s41586-024-07620-9 38961290 PMC11254748

[101] Fendt SM. 2024. 100 years of the Warburg effect: a cancer metabolism endeavor. Cell 187:3824–3828. doi:10.1016/j.cell.2024.06.026 39059359

[102] Lebowitz PJ, Schwartzberg LS, Bruce AK. 1976. The *in vivo* association of manganese with the chromosome of *Micrococcus radiodurans*. Photochem Photobiol 23:45–50. doi:10.1111/j.1751-1097.1976.tb06769.x 178008

[103] Ma C, Bloomfield VA. 1994. Condensation of supercoiled DNA induced by MnCl2. Biophys J 67:1678–1681. doi:10.1016/S0006-3495(94)80641-1 7819499 PMC1225529

[104] Chen P, Chakraborty S, Peres TV, Bowman AB, Aschner M. 2015. Manganese-induced neurotoxicity: from *C. elegans* to humans. Toxicol Res (Cambridge) 4:191–202. doi:10.1039/c4tx00127c 25893090 PMC4399965

[105] Nicolai MM, Weishaupt AK, Baesler J, Brinkmann V, Wellenberg A, Winkelbeiner N, Gremme A, Aschner M, Fritz G, Schwerdtle T, Bornhorst J. 2021. Effects of manganese on genomic integrity in the multicellular model organism *Caenorhabditis elegans.* Int J Mol Sci 22:10905. doi:10.3390/ijms222010905 34681565 PMC8535284

[106] Garg R, David MS, Yang S, Culotta VC. 2024. Metals at the host–fungal pathogen battleground. Annu Rev Microbiol 78:23–38. doi:10.1146/annurev-micro-041222-023745 38781605 PMC12044431

[107] Hammond NG, Cameron RB, Faubert B. 2024. Beyond glucose and Warburg: finding the sweet spot in cancer metabolism models. NPJ Metab Health Dis 2:11. doi:10.1038/s44324-024-00017-2 40603611 PMC12118702

[108] Daly MJ, Ouyang L, Fuchs P, Minton KW. 1994. *In vivo* damage and *recA*-dependent repair of plasmid and chromosomal DNA in the radiation-resistant bacterium *Deinococcus radiodurans*. J Bacteriol 176:3508–3517. doi:10.1128/jb.176.12.3508-3517.1994 8206827 PMC205538

[109] Daly MJ, Minton KW. 1996. An alternative pathway of recombination of chromosomal fragments precedes *recA*-dependent recombination in the radioresistant bacterium *Deinococcus radiodurans*. J Bacteriol 178:4461–4471. doi:10.1128/jb.178.15.4461-4471.1996 8755873 PMC178212

[110] Zückert WR. 2007. Laboratory maintenance of *Borrelia burgdorferi*. Curr Protoc Microbiol Chapter 12:1–10. doi:10.1002/9780471729259.mc12c01s4 18770608

[111] Stoll S, Schweiger A. 2006. EasySpin, a comprehensive software package for spectral simulation and analysis in EPR. J Magn Reson 178:42–55. doi:10.1016/j.jmr.2005.08.013 16188474

[112] Davoust CE, Doan PE, Hoffman BM. 1996. Q-band pulsed electron spin-echo spectrometer and its application to ENDOR and ESEEM. J Magn Reson A 119:38–44. doi:10.1006/jmra.1996.0049

[113] Epel B, Gromov I, Stoll S, Schweiger A, Goldfarb D. 2005. Spectrometer manager: a versatile control software for pulse EPR spectrometers. Concepts Magn Reson 26B:36–45. doi:10.1002/cmr.b.20037

[114] Doan PE, Hoffman BM. 1997. Making hyperfine selection in Mims ENDOR independent of deadtime. Chem Phys Lett 269:208–214. doi:10.1016/S0009-2614(97)00293-5

[115] Schweiger A, Jeschke G. 2001. Principles of pulse electron paramagnetic resonance. Oxford University Press.

