# Supplementary material for: EPR spectroscopy reveals antioxidant manganese defenses in the Lyme disease pathogen Borrelia burgdorferi
Source: mBio. 2025 Nov 13;16(12):e02824-25. doi: 10.1128/mbio.02824-25 (PMC12691683; doi:10.1128/mbio.02824-25)
Supplement: Supplemental material — Texts S1 and S2 and legends for Fig. S1-S6. [file mbio.02824-25-s0007.docx]

**SUPPLEMENTAL TEXT 1. Strategic Localization of Antioxidant Enzymes in Bacteria.**

Most microorganisms rely on cytoplasmic MnSOD (SodA) and Fe-catalase (KatE) enzymes to detoxify O_2_^•−^ and H_2_O_2_, respectively. However, some bacteria—such as *D. radiodurans*, *L. plantarum*, and *B. subtilis*—accumulate H-Mn antioxidants that provide protection against ROS damage to the Fe-proteome, especially under conditions where H₂O₂ enters the cytoplasm from the extracellular environment (1, 2).

In *D. radiodurans*, the constitutively expressed MnSOD (DR1279) and Fe-catalase (DR1998) lack typical signal peptides or TAT leaders and are synthesized in the cytoplasm (3, 4). Nevertheless, mounting evidence suggests that these enzymes act at the cell boundary. For example, *D. radiodurans* mutants lacking MnSOD (*sodA*^-^) are hypersensitive to exogenous superoxide generators such as paraquat, as well as desiccation-sensitive, despite being fully resistant to acute and chronic γ-radiation (5). Similarly, *D. radiodurans* catalase (KatE) is essential for resistance to high-level chronic γ-irradiation––but KatE is dispensable for resistance to acute γ-irradiation. This suggests a protective role of Fe-catalase at or near the cell boundary (6).

Proper localization of antioxidant enzymes allows bacteria to respond to extracellular ROS. While most proteins are synthesized in the cytoplasm, over one-third are ultimately directed to extracytoplasmic compartments (e.g., the periplasm) via the SecA or signal recognition particle (SRP) pathways (7, 8). In *D. radiodurans*, both the SRP pathway and numerous small RNAs contribute to stress survival and the trafficking of antioxidant enzymes (9). Significantly, co-culture of *D. radiodurans* and *E. coli* as mixed colony biofilms on plates confers extreme resistance to chronic γ-irradiation (Cs-137) on the typically-radiosensitive *E. coli*. This finding supports the conclusion that catalase in *D. radiodurans* must act at or near the cell surfaces in contact with *E. coli* (6).

Finally, Irwin Fridovich and colleagues first suggested that environmental O_2_^•−^ assault at the cell surface should not create a need for intracellular SOD activity in bacteria (10, 11). *B. burgdorferi* encodes components of both the SecA and SRP pathways (8), raising the possibility that MnSOD or other enzymes (12) may be trafficked to outer compartments including the periplasm in this spirochete, which is significantly larger than *E. coli*. It is not conceivable to us that an extremely auxotrophic Fe-independent large bacterium would dedicate a significant fraction of its MnSOD inventory to protecting its Fe-free cytoplasm, which is sealed off from O_2_^•−^ by the inner membrane. We thus assign MnSOD to the periplasm (**Fig. 8**, main text).

**SUPPLEMENTAL TEXT 2. Mechanism of Superoxide Toxicity in Fe‑Free B. burgdorferi.**

The toxicity of O_2_^•−^ in irradiated B. burgdorferi, an organism essentially devoid of redox‑active iron, is mechanistically intriguing—O_2_^•−^ radicals must be targeting cellular components other than Fe‑dependent enzymes. In this study, B. burgdorferi cells were γ‑irradiated in the frozen state (−79 °C), a condition that preserves O_2_^•−^ and H₂O₂ in the ice matrix but not short‑lived hydroxyl radicals (^•^OH). Upon thawing, the Fe-free spirochetes are transiently exposed to a pulse of H₂O₂ and O_2_^•−^, simulating the oxidative burst generated by the host innate immune system (**Fig. 1**, main text). While H₂O₂ is freely permeable and diffuses throughout the cell, O_2_^•−^ does not cross membranes readily (13), so damage is concentrated at the cell periphery, including membrane‑bound outer surface proteins (OSPs) and the periplasm (14, 15). The inner membrane forms the final barrier to O_2_^•−^ entering the cytoplasm (**Fig. 8**). Thus, in B. burgdorferi, O_2_^•−^ generated by γ-rays likely harms Fe‑independent processes by several mechanisms:

1. **Direct oxidation of amino acids in proteins.** In addition to its well‑known inactivation of Fe–S enzymes, O_2_^•−^ can directly oxidize amino acids leading to protein carbonylation and functional impairment. In both *in vivo* and *in vitro* settings, O_2_^•−^ shows minimal reactivity toward DNA, making proteins the primary target (2, 15, 16). This mechanistic distinction underlies the success of γ‑irradiation to inactivate pathogen genomes for vaccine production while leaving protein epitopes undamaged (17–20). Synthetic H‑Mn antioxidants such as MDP protect proteins and lipids in whole‑cell and whole‑virus preparations from O_2_^•−^‑dependent oxidation even at supra‑lethal γ‑ray doses (>60 kGy) which nonetheless destroy DNA and RNA genomes by radiolytic ^•^OH (2, 20, 21).
2. **Oxidative damage to Cu‑dependent proteins.** Although B. burgdorferi relies on Mn rather than Fe for proteome maintenance, it encodes Cu‑dependent enzymes (e.g., BicA) essential for survival and infectivity (22, 23). As with Fe, Cu can participate in redox-cycling that promotes ROS formation. The redox‑active Cu centers in these proteins are therefore plausible targets for O_2_^•−^ attack, necessitating protection by MnSOD or H‑Mn antioxidants.
3. **Disruption of Mn redox-cycling with increasing Mn²⁺ levels.** At low‑mM concentrations, Mn²⁺ can catalytically scavenge O_2_^•−^ without releasing ^•^OH, producing H₂O₂ and O₂ (24). In the presence of orthophosphate (Pi), Mn²⁺ forms simple Mn–Pi complexes with enhanced activity (17), and the dismutation rate constants for O_2_^•−^ increase with Mn²⁺ concentration accordingly (25). However, under γ‑irradiation, high‑mM Mn²⁺ concentration can disrupt Mn(II)/(III) redox-cycling, generating Mn(III)/Mn(IV) oxides that are strong oxidants of proteins and nucleic acids (24). We suggest that in metabolite‑limited B. burgdorferi that have hyperaccumulated Mn²⁺, γ‑irradiation may transiently generate Mn³⁺/Mn⁴⁺ species capable of mismetalating and oxidizing the Mn‑proteome, and damaging DNA (main text).

**SUPPLEMENTAL REFERENCES**

1. Brim H, Osborne JP, Kostandarithes HM, Fredrickson JK, Wackett LP, Daly MJ. 2006. *Deinococcus radiodurans* engineered for complete toluene degradation facilitates Cr(VI) reduction. Microbiology (N Y) 152:2469–2477.

2. Daly MJ. 2023. The scientific revolution that unraveled the astonishing DNA repair capacity of the Deinococcaceae: 40 years on. Can J Microbiol 69:369–386.

3. Makarova KS, Aravind L, Wolf YI, Tatusov RL, Minton KW, Koonin E V., Daly MJ. 2001. Genome of the extremely radiation-resistant bacterium *Deinococcus radiodurans* viewed from the perspective of comparative genomics. Microbiology and Molecular Biology Reviews 65.

4. Lipton MS, Pǎá-Toli L, Anderson GA, Anderson DJ, Auberry DL, Battista JR, Daly MJ, Fredrickson J, Hixson KK, Kostandarithes H, Masselon C, Markillie LM, Moore RJ, Romine MF, Shen Y, Stritmatter E, Tolić N, Udseth HR, Venkateswaran A, Wong KK, Zhao R, Smith RD. 2002. Global analysis of the *Deinococcus radiodurans* proteome by using accurate mass tags. Proc Natl Acad Sci U S A 99:11049.

5. Gaidamakova EK, Sharma A, Matrosova VY, Grichenko O, Volpe RP, Tkavc R, Conze IH, Klimenkova P, Balygina I, Horne WH, Gostincar C, Chen X, Makarova KS, Shuryak I, Srinivasan C, Jackson-Thompson B, Hoffman BM, Daly MJ. 2022. Small-molecule Mn antioxidants in *Caenorhabditis elegans* and *Deinococcus radiodurans* supplant MnSOD enzymes during aging and irradiation. mBio 13:e0339421.

6. Shuryak I, Matrosova VY, Gaidamakova EK, Tkavc R, Grichenko O, Klimenkova P, Volpe RP, Daly MJ. 2017. Microbial cells can cooperate to resist high-level chronic ionizing radiation. PLoS One 12:e0189261.

7. Weiner JH, Li L. 2008. Proteome of the *Escherichia coli* envelope and technological challenges in membrane proteome analysis. Biochim Biophys Acta Biomembr 1778:1698–1713.

8. Zückert WR. 2019. Protein Secretion in Spirochetes. Microbiol Spectr 7.

9. Han R, Fang J, Jiang J, Gaidamakova EK, Tkavc R, Daly MJ, Contreras LM. 2020. Signal recognition particle RNA contributes to oxidative stress response *in Deinococcus radiodurans* by modulating catalase localization. Front Microbiol 11.

10. Lynch RE, Fridovich I. 1978. Permeation of the erythrocyte stroma by superoxide radical. Journal of Biological Chemistry 253.

11. Liochev SI, Fridovich I. 2005. Cross-compartment protection by SOD1. Free Radic Biol Med 38.

12. Dutta S, Rana VS, Backstedt BT, Shakya AK, Kitsou C, Yas OB, Smith AA, Ronzetti MH, Lipman RM, Araujo-Aris S, Yang X, Rai G, Lin YP, Herzberg O, Pal U. 2025. *Borrelial* phosphomannose isomerase as a cell surface localized protein that retains enzymatic activity and promotes host-pathogen interaction. mBio 16.

13. Imlay JA. 2025. The barrier properties of biological membranes dictate how cells experience oxidative stress. Mol Microbiol 123:454–463.

14. Esteve-Gassent MD, Smith TC, Small CM, Thomas DP, Seshu J. 2015. Absence of sodA increases the levels of oxidation of key metabolic determinants of *Borrelia burgdorferi*. PLoS One 10.

15. Londoño AF, Sharma A, Sealy J, Rana VS, Foor SD, Matrosova VY, Gaidamakova EK, Volpe RP, Daly MJ, Hoffman BM, Pal U, Dumler JS. 2025. *Borrelia burgdorferi* radiosensitivity and Mn antioxidant content: antigenic preservation and pathobiology. mBio 16.

16. Daly MJ. 2012. Death by protein damage in irradiated cells. DNA Repair (Amst) 11:12–21.

17. Daly MJ, Gaidamakova EK, Matrosova VY, Kiang JG, Fukumoto R, Lee DY, Wehr NB, Viteri GA, Berlett BS, Levine RL. 2010. Small-molecule antioxidant proteome-shields in *Deinococcus radiodurans*. PLoS One 5:10–15.

18. Gaidamakova EK, Myles IA, McDaniel DP, Fowler CJ, Valdez PA, Naik S, Gayen M, Gupta P, Sharma A, Glass PJ, Maheshwari RK, Datta SK, Daly MJ. 2012. Preserving immunogenicity of lethally irradiated viral and bacterial vaccine epitopes using a radio- protective Mn^2+^-peptide complex from *Deinococcus*. Cell Host Microbe 12:117–124.

19. Tobin GJ, Tobin JK, Gaidamakova EK, Wiggins TJ, Bushnell R V., Lee WM, Matrosova VY, Dollery SJ, Meeks HN, Kouiavskaia D, Chumakov K, Daly MJ. 2020. A novel gamma radiation-inactivated sabin-based polio vaccine. PLoS One 15:e0228006.

20. Broder KC, Matrosova VY, Tkavc R, Gaidamakova EK, Ho LTVT, Macintyre AN, Soc A, Diallo A, Darnell SC, Bash S, Daly MJ, Jerse AE, Liechti GW. 2024. Irradiated whole cell *Chlamydia* vaccine confers significant protection in a murine genital tract challenge model. NPJ Vaccines 9.

21. Yang H, Sharma A, Daly MJ, Hoffman BM. 2024. The ternary complex of Mn^2+^, synthetic decapeptide DP1 (DEHGTAVMLK), and orthophosphate is a superb antioxidant. Proc Natl Acad Sci U S A 121:2417389121.

22. Wang P, Lutton A, Olesik J, Vali H, Li X. 2012. A novel iron- and copper-binding protein in the Lyme disease spirochaete. Mol Microbiol 86:1441–1451.

23. Wang P, Yu Z, Santangelo TJ, Olesik J, Wang Y, Heldwein E, Li X. 2017. BosR is a novel Fur family member responsive to copper and regulating copper homeostasis in *Borrelia burgdorferi*. J Bacteriol 199.

24. Daly MJ, Gaidamakova EK, Matrosova VY, Vasilenko A, Zhai M, Leapman RD, Lai B, Ravel B, Li SMW, Kemner KM, Fredrickson JK. 2007. Protein oxidation implicated as the primary determinant of bacterial radioresistance. PLoS Biol 5:769–779.

25. Barnese K, Gralla EB, Valentine JS, Cabelli DE. 2012. Biologically relevant mechanism for catalytic superoxide removal by simple manganese compounds. Proc Natl Acad Sci U S A 109.

**SUPPLEMENTAL FIGURE LEGENDS (S1-S6)**

**FIG. S1**: 2K, 35 GHz 2K Pulsed EPR spectra. Partitioning of Mn^2+^ EPR spectra of WT and *sodA^-^ Day-0 Borrelia* cells into contributions from H-Mn and L-Mn. See Materials and Methods for details.

**FIG. S2**: 2K, 35 GHz 2K absorption display CW EPR spectra. Partitioning of Mn^2+^ EPR spectra of WT *Day-0 Borrelia* cells with and without Mn-supplementation into contributions from H-Mn and L-Mn. See Materials and Methods for details. Numbers are the integrated intensity of partitioned H and L EPR spectra and proportional to relative Mn^2+^ concentration.

**FIG. S3**: 2K, 35 GHz 2K absorption display CW EPR spectra. Partitioning of Mn^2+^ EPR spectra of *sodA^-^ Day-0 Borrelia* cells with and without Mn-supplementation into contributions from H-Mn and L-Mn. See M&M for details. Numbers are the integrated intensity of partitioned H and L EPR spectra and proportional to relative Mn^2+^ concentration.

**FIG. S4**: 2K, 35 GHz 2K absorption display CW EPR spectra. Partitioning of Mn^2+^ EPR spectra of WT *Day-4 Borrelia* cells with and without Mn-supplementation into contributions from H-Mn and L-Mn. See Materials and Methods for details. Numbers are the integrated intensity of partitioned H and L EPR spectra and proportional to relative Mn^2+^ concentration. Experimental and H-Mn spectrum amplitude is shrunk to ½ of its actual height (×0.5).

**FIG. S5**: 2K, 35 GHz 2K absorption display CW EPR spectra. Partitioning of Mn^2+^ EPR spectra of *sodA^-^ Day-6 Borrelia* cells with and without Mn-supplementation into contributions from H-Mn and L-Mn. See Materials and Methods for details. Numbers are the integrated intensity of partitioned H and L EPR spectra and proportional to relative Mn^2+^ concentration.

**FIG. S6**: *Borrelia* antioxidants H-Mn have no nitrogenous ligands bound. 35 GHz 2K, 3-pulse ^14^N ESEEM spectra of Mn-supplemented *B. burgdorferi* WT/ΔMnSOD for Day-0 and Day-4/6 time-points (see main text). Mn-imidazole (1:10) frozen solution show ^14^N modulation due to bound imidazole, whereas all borrelia samples lack any ^14^N signal and thus no bound nitrogenous ligands. See Materials and Methods for details.
